# MIR222HG attenuates macrophage M2 polarization and allergic inflammation in allergic rhinitis by targeting the miR146a-5p/TRAF6/NF-κB axis

**DOI:** 10.3389/fimmu.2023.1168920

**Published:** 2023-05-02

**Authors:** Silu Wen, Fen Li, Yulei Tang, Lin Dong, Yan He, Yuqin Deng, Zezhang Tao

**Affiliations:** ^1^ Department of Otolaryngology-Head and Neck Surgery, Renmin Hospital of Wuhan University, Wuhan, Hubei, China; ^2^ Department of Otolaryngology, First College of Clinical Medical Science, Wuhan University, Wuhan, Hubei, China; ^3^ Institute of Otolaryngology-Head and Neck Surgery, Renmin Hospital of Wuhan University, Wuhan, Hubei, China

**Keywords:** allergic rhinitis, long non-coding RNAs, miR146a-5p, TRAF6, macrophages polarization

## Abstract

Although M2 macrophages are involved in the orchestration of type 2 inflammation in allergic diseases, the mechanisms underlying non-coding RNA (ncRNA)-mediated macrophage polarization in allergic rhinitis (AR) have not been systematically understood. Here, we identified long non-coding RNA (lncRNA) MIR222HG as a key regulator of macrophage polarization and revealed its role in AR. Consistent with our bioinformatic analysis of GSE165934 dataset derived from the Gene Expression Omnibus (GEO) database, lncRNA-MIR222HG and murine mir222hg were downregulated in our clinical samples and animal models of AR, respectively. Mir222hg was upregulated in M1 macrophages and downregulated in M2 macrophages. The allergen-ovalbumin facilitated polarization of RAW264.7 cells to the M2 phenotype, accompanied by the downregulation of mir222hg expression in a dose-dependent manner. Mir222hg facilitates macrophage M1 polarization and reverses M2 polarization caused by ovalbumin. Furthermore, mir222hg attenuates macrophage M2 polarization and allergic inflammation in the AR mouse model. Mechanistically, a series of gain- and loss-of-function experiments and rescue experiments were performed to verify the role of mir222hg as a ceRNA sponge that adsorbed miR146a-5p, upregulated Traf6, and activated the IKK/IκB/P65 pathway. Collectively, the data highlight the remarkable role of MIR222HG in the modulation of macrophage polarization and allergic inflammation, as well as its potential role as a novel AR biomarker or therapeutic target.

## 1 Introduction

Allergic rhinitis (AR) is an immunoglobulin E (IgE)-mediated, heterogeneous, chronic nasal condition experienced by allergen-sensitized individuals and characterized by symptoms, such as sneezing, nasal obstruction, pruritus, and rhinorrhea (1). In sensitized individuals, a dysregulated type 2 immune response can be triggered in AR (2). Recently, M2 macrophages have been found to amplify the type 2 immune response by stimulating T helper 2 (Th2) cells, eosinophils, and basophils to release cytokines and chemokines, including IL-13, CCL17, CCL22, and CCL24, which could activate Th2 cells, promote accumulation of eosinophils, and aggravate allergic Th2 inflammation (3–7). Although researchers have achieved great advances in allergen-specific immunotherapies in the past several years (8), these treatments have failed to meet the levels of efficacy expected by both clinicians and patients. However, RNA-based therapeutics are currently in the preclinical stages of development and show remarkable promise as novel agents and treatments (9).

Non-coding RNA (ncRNA) is a class of RNAs that does not code for protein but may affect normal gene expression. Depending on their size, ncRNAs can be divided into two main subtypes: small ncRNA (for example, miRNAs and small nuclear RNA) and long non-coding RNAs (lncRNAs) (> 200 bp) (10, 11). Some lncRNA genes harbor miRNA genes in their gene loci and are therefore categorized as miRNA-host-gene-derived lncRNAs (12). MIR222HG, a little-known miRNA-host-gene-derived lncRNA, is the host transcript for the miR-221 and miR-222 genes (13).

lncRNAs typically display lower levels of expression than protein-coding messenger RNAs (mRNAs); however, their expression is stronger in tissues where they are commonly found, consistent with the established roles of lncRNAs in the regulation of cell-type-specific processes (14, 15). Genome-wide studies have determined that lncRNAs can markedly influence the type, duration, and magnitude of macrophage-mediated immune response (16–18). However, the molecular mechanisms responsible for the association of ncRNAs with macrophages in AR remain unclear.

In this study, we performed an in silico microarray analysis of previously published data from the Gene Expression Omnibus (GEO) database (GSE165934, https://www.ncbi.nlm.nih.gov/geo/) and found that lncRNA-MIR222HG was downregulated in allergic patients with asthma. Furthermore, we validated our bioinformatic analysis by performing real-time quantitative polymerase chain reaction (qRT-PCR), which revealed that MIR222HG and murine mir222hg were downregulated in our clinical AR samples (39 cases of AR and 40 controls) and animal models of AR (10 AR mice and 10 controls), respectively. Our data demonstrated MIR222HG can act as a competitive endogenous RNA (ceRNA) sponge (19), compete with miR146a-5p, and activate the TRAF6/IKK/IκB/P65 signaling pathway, thus regulating macrophage polarization, and consequently, AR pathogenesis. The findings of our study could facilitate ongoing ncRNA-mediated macrophage polarization research on AR and provide novel therapeutic targets.

## 2 Materials and methods

### 2.1 Bioinformatic analyses

Gene expression profiles from the GSE165934 dataset were acquired from the NCBI GEO database. The GSE165934 dataset contains 10 allergic asthma patients’ peripheral blood mononuclear cells (PBMCs) gene expression profiles and those of nine healthy controls. The GSE165934 dataset and its annotation information from the GPL23126 platform were downloaded and analyzed using R (version 4.1.0, R Foundation for Statistical Computing, Vienna, Austria) and R Studio software. Average RNA expression values were used when duplicate data was obtained. Hub differentially expressed genes (DEGs) were annotated using the limma package (20). Our screening criteria for DEGs were |log2FC| ≥ 1 with P-value < 0.05. Heatmap and volcano plot analyses were performed using the R software to better visualize the DEGs.

### 2.2 Construction of the ceRNA network

Based on data from associated annotation files, DEGs were separated into DE-lncRNAs, DE-miRNAs, and DE-mRNAs. According to conservation analyses by NONCODE (http://www.noncode.org/), UCSC (http://genome.ucsc.edu/), Ensembl (http://asia.ensembl.org/index.html), and LNCipedia (https://lncipedia.org/) databases, we selected conserved DE-lncRNAs for further analysis and regarded them as hub lncRNAs (hub DE-lncRNAs) (21). In terms of log_2_FC values, the top 10 DE-miRNAs were identified as hub DE-miRNAs and the top 20 DE-mRNAs were regarded as hub DE-mRNAs (21). Hub DE-lncRNA – hub DE-miRNA – DE-mRNA interactions were predicted using LncBase (https://diana.e-ce.uth.gr/lncbasev3/home), TargetScan (https://www.targetscan.org/vert_80/), miRDB (http://mirdb.org/), and TarBase (http://www.microrna.gr/tarbase) databases (22). The ceRNA network formed by lncRNA-miRNA-mRNA connection was then integrated using the Cytoscape software (version 3.9.1) and visualized using a Sankey diagram (23).

### 2.3 Enrichment analyses and immune cell composition analyses

Kyoto Encyclopedia of Genes and Genomes (KEGG) and Gene Ontology (GO) enrichment analyses were used to explore the potential biological functions of DE-mRNAs (24).

The CIBERSORT algorithm was applied to quantify the ratios of 22 types of immune cells in PBMCs of patients with allergic asthma and healthy controls through the transcriptome expression matrix. Mann–Whitney U test was used for comparisons between the two groups and matrix correlation analyses was utilized to investigate the correlation between the expression of four highly conserved hub DE-lncRNAs and the ratios of immune cells.

The graphical output of the program was provided by the Sangerbox software (http://vip.sangerbox.com/).

### 2.4 Patients and clinical specimens

A total of 79 patients (39 with AR and 40 controls) were recruited from the Otorhinolaryngology Department of the Renmin Hospital of Wuhan University, Wuhan, China. The detailed clinical characteristics of the enrolled patients are presented in the **Table 1**. The diagnostic criteria for AR were based on Allergic Rhinitis and its impact on asthma guidelines (25). Patients who had clinical histories and positive skin prick tests or allergen-specific IgE ≥ 0.7 kU/L (ImmunoCAP 250 allergen detection system) were included in the AR group (n = 39); patients with allergen-specific IgE < 0.35 IU/mL were classified as the control group (n = 40) (26). Subjects with infectious nasal diseases, severe nasal septum deviations, tumors, and systemic diseases were excluded. No drugs were administered for two weeks before allergen-specific IgE detection. Five milliliters of venous blood were collected from each patient in an EDTA-anticoagulated tube (Becton, Dickinson and Company, NJ, USA). We used lymphocyte separation medium (TBDscience, Tianjin, China) to isolate PBMCs *via* density gradient centrifugation.

**Table 1:** Clinical characteristics of enrolled patients.

		Control	AR
Subjects (n)		40	39
Age (year)		27.90±18.54	24.37±16.29
Gender (Male/Female)		22 / 18	16 / 23
History (year)		0	3.48±1.21
Dermatophagoides farinae (+,n)		0	39
House dust mite (+,n)		0	38
Animal dander (+,n)		0	4
Allergen-specific IgE(IU/ml)	Dermatophagoides farinae	0.04±0.02	72.27±36.97
	House dust mite	0.02±0.01	63.12±28.32
	Animal dander	0	2.64±1.98

The mean ± SD are shown.

### 2.5 Animal models and ethical approval

Specific-Pathogen-Free (SPF) BALB/C mice (10 male, 10 female) aged six weeks were purchased from the Vital River Laboratory Animal Technology (Beijing, China) (License No.: SCXK2016-0006) and raised in an SPF barrier environment at the Animal Experiment Center of the Renmin Hospital of Wuhan University with food and water ad libitum and a regular light-dark cycle (License No.: SYXK2015-0027).

The AR animal model was established as previously reported (27). Mice were randomly divided into two groups, i.e., control (5 male, 5 female) and AR (5 male, 5 female) groups. To avoid the breeding of mice, female and male mice were raised in separate cages. To achieve basic sensitization, 200 μL of the suspensions of 100 μg ovalbumin (OVA, Sigma-Aldrich, MO, USA, A5503) and 4 mg Al(OH)_3_ in saline was intraperitoneally injected on days 0, 7, and 14 in the AR group (n = 10). On days 21–27, 20 μL of saline supplemented with 800 μg OVA was administered intranasally, in each side of the nasal cavity (10 μL per nasal cavity), once a day for 1 week. The control group (n = 10) was administered a vehicle control, saline. Within 30 min of the last nasal challenge, allergic symptoms, such as sneezing, runny nose, and scratching, were consistently elicited in the AR group and subsequently quantified to evaluate the incidence and severity of AR symptoms (28). Mice were euthanized for subsequent experiments. Nasal mucosa was embedded in paraffin, sectioned, and then stained with hematoxylin and eosin (HE) and periodic acid-Schiff (PAS) (29). The mice spleens were obtained and stored at -80°C for qRT-PCR. Details regarding the animal model are provided in the **Supplementary Figure S1**.

### 2.6 Cell culture and stimulation

Murine RAW264.7 monocyte-macrophages, human THP-1 monocytes, and human HEK293T cells were purchased from the Cell Bank of the China Academy of Sciences (Shanghai, China). RAW264.7, and HEK293T cells were cultured in Dulbecco’s modified Eagle medium-high glucose (HyClone, UT, USA) containing 10% Fetal Bovine Serum (Gibco, CA, USA) at 37°C in an atmosphere of 5% CO_2_ and 95% air (30). THP-1 was cultured in RPMI-1640 medium (HyClone, UT, USA), supplemented with 10% Fetal Bovine Serum (Gibco, CA, USA), antibiotics (streptomycin 0.1 mg/mL and penicillin 100 U/mL) (30). Only the logarithmic growth phase cells were selected for experiments in the present study.

Control and transfected RAW264.7 cells were induced to the M1 macrophage following treatment with 100 ng/mL LPS (Sigma-Aldrich, MO, USA, L4391) for 24 h and M2 macrophage following treatment with 40 ng/mL IL-4 (PeproTech, NJ, USA) for 48 h (30). Cells were further treated with various concentrations of OVA (0.1–500 μg/mL; Sigma-Aldrich, MO, USA, A5503) for 48 h to evaluate the effect of OVA on macrophage polarization.

### 2.7 Oligonucleotides *in vitro* Transfection

Small interfering RNAs of mir222hg (si-mir222hg-1–5), miR146a-5p mimics, miR146a-5p inhibitor, and their respective controls were obtained from Ribobio (Guangzhou, Guangdong, China). The above-mentioned oligonucleotides were transfected using Advanced RNA Transfection Reagent (ZetaLife, CA, USA, AD600075) in accordance with the manufacturer’s protocol (31). In brief, RAW264.7 cells were seeded into six-well culture dish at a density of 10^5^ cells/well and incubated as above before transfection. Twenty-four hours after plating, the culture media was replaced with Advanced RNA Transfection Reagent and oligonucleotide mixture. Eight hours after transfection, the percentage of FAM/Cy3-positive cells was measured and counted under an inverted fluorescence microscope (Olympus, Tokyo, Japan, IX73). Twenty-four hours after transfection, we replaced the medium with fresh culture medium, and then added LPS/IL-4/OVA at the given dose. qRT-PCR was performed to verify transfection efficiency. Oligonucleotide sequences are listed in the **Table 2**.

**Table 2:** List of oligonucleotide sequences used for transfection.

Gene Name		Sequence(5’-3’)
si-mir222hg-1	sense	CAGAUCAAGCCUAGUACCUUUTT
	antisense	AAAGGUACUAGGCUUGAUCUGTT
si-mir222hg-2	sense	CCCAAUCGAUUCACUAUUCAUTT
	antisense	AUGAAUAGUGAAUCGAUUGGGTT
si-mir222hg-3	sense	GGAUACUGCUCAGUAAUGUAATT
	antisense	UUACAUUACUGAGCAGUAUCCTT
si-mir222hg-4	sense	UUGAAGCCGAAAGCUUCUAAATT
	antisense	UUUAGAAGCUUUCGGCUUCAATT
si-mir222hg-5	sense	UGCAAGUUAAUGGGUGAACAATT
	antisense	UUGUUCACCCAUUAACUUGCATT
miR146a-5p mimic	sense	UGAGAACUGAAUUCCAUGGGUU
	antisense	ACUCUUGACUUAAGGUACCCAA
miR146a-5p inhibitor	–	AACCCAUGGAAUUCAGUUCUCA

### 2.8 Vector construction and transfection

Mir222hg–overexpressing lentivirus (mir222hg LV) and NC LV were synthesized by GeneChem (Shanghai, China). RAW264.7 cell lines stably overexpressing mir222hg were constructed according to the manufacturer’s protocol (32, 33). Seventy-two hours after transfection, the percentage of EGFP-positive cells was measured and counted under an inverted fluorescence microscope. Stably transfected RAW264.7 cells were then cultured in media containing 2 μg/mL puromycin (MCE, NJ, USA) for 2 weeks to isolate successful transfections. qRT-PCR was performed to verify transfection efficiency.

### 2.9 Animal model treated with mir222hg-overexpressing lentivirus

Thirty-two female BALB/C mice were randomly divided into four groups (n= 8 in each group): the Control group, AR group, AR + NC LV group, and AR + mir222hg LV group. The AR animal model was established as described above. Mice in the AR + NC LV group and AR + mir222hg LV groups were intranasally administered 2×10^6^ TU of mir222hg-overexpressing lentivirus or negative control lentivirus two days before day 21, 23, 25, and 27 (34). Mice were euthanized for subsequent experiments. Nasal mucosa were embedded in paraffin, sectioned, and then stained with HE and PAS. The mice spleens were harvested and ground as single-cell suspensions. Macrophages were isolated from single-cell suspensions using differential adhesion.

### 2.10 Real-Time Quantitative Polymerase Chain Reaction

Total RNA was isolated using TRIzol (Invitrogen, CA, USA,15596026), and the resultant RNA purity and concentration were measured using a NanoDrop spectrophotometer (Thermo Fisher Scientific, MA, USA, ND2000). Briefly, an Evo M-MLV RT Mix Kit (Accurate Biotechnology, Hunan, China, AG11705) was used to synthesize complementary DNA. miRNA was reverse transcribed into complementary DNA using the miRNA 1st strand complementary DNA synthesis kit (Accurate Biotechnology, Hunan, China, AG11717). A SYBR^®^ Green Premix Pro Taq HS qPCR Kit (Accurate Biotechnology, Hunan, China, AG11701) was used to perform qRT-PCR on a Bio-Rad CFX Real-time PCR system (Bio-Rad, CA, USA). β-Actin and U6 were used as internal references for mRNA and miRNA transcript-levels quantification, respectively. Absolute qRT-PCR was performed as reported previously (35). Standard curves of mir222hg and miR146a-5p were generated *via* the serial dilution of standard mir222hg (Tsingke Biotechnology, Beijing, China) and standard miR146a-5p (Accurate Biotechnology, Hunan, China). Data are presented as copies per 100 pg total RNA (2×10^5^ cells). The primers were synthesized by Sangon Biotech (Shanghai, China) (32). The primer sequences for all genes are provided in **Table 3**.

**Table 3:** List of primers used for qRT-PCR analysis.

Genes	Spezies	Primer	Primer sequence(5’–3’)
AC092747.4	human	Forward primer	ACACCAGTCTCTCAATAGGG
		Reverse primer	CTCCTCCCAAACTCCATAGG
SMIM30	human	Forward primer	TTGGTGCTGCCTGTTGTGGAAG
		Reverse primer	CAAATGCCTGTAATGCTGAGAACCAC
AC234772.1	human	Forward primer	CGTCTTCATTCAGGAGGGG
		Reverse primer	GGAAGTAGGTAGGTTTAACAGGTC
MIR222HG	human	Forward primer	AGGCTGGTGTGGTAAAGGGA
		Reverse primer	AAGGCATAGCACCCACAAGC
β-ACTIN	human	Forward primer	CCTGGCACCCAGCACAAT
		Reverse primer	GGGCCGGACTCGTCATAC
ac092747.4	mouse	Forward primer	GCCTCAAACTGGGATAAAGG
		Reverse primer	CCAGACTTAGAAGGACTTGCAG
smim30	mouse	Forward primer	AGTCCTCGCTTCGCTGCTTTTG
		Reverse primer	CACATCTGTCCATTTCGCTTTCTTGC
ac234772.1	mouse	Forward primer	CAGGTTTGTGGTGGAATGGAC
		Reverse primer	GGTGTCCCTTTCTCCCTTAAC
mir222hg	mouse	Forward primer	GATGCCTGGAAAATGACTCTG
		Reverse primer	GGTCCTTCTTGTATGTTCCC
IL-1β	mouse	Forward primer	GAAATGCCACCTTTTGACAGTG
		Reverse primer	TGGATGCTCTCATCAGGACAG
TNF-α	mouse	Forward primer	GTTCTATGGCCCAGACCCTCAC
		Reverse primer	GGCACCACTAGTTGGTTGTCTTTG
iNOS	mouse	Forward primer	GTTCTCAGCCCAACAATACAAGA
		Reverse primer	GTGGACGGGTCGATGTCAC
ARG1	mouse	Forward primer	AGGACAGCCTCGAGGAGGGG
		Reverse primer	CCTGGCGTGGCCAGAGATGC
PPAR-γ	mouse	Forward primer	TTCGATCCGTAGAAGCCGTG
		Reverse primer	TTGGCCCTCTGAGATGAGGA
IL-10	mouse	Forward primer	CAGTACAGCCGGGAAGACAA
		Reverse primer	CCTGGGGCATCACTTCTACC
YM1	mouse	Forward primer	CATGAGCAAGACTTGCGTGAC
		Reverse primer	GGTCCAAACTTCCATCCTCCA
CD86	mouse	Forward primer	CATGGGCTTGGCAATCCTTA
		Reverse primer	AAATGGGCACGGCAGATATG
CD206	mouse	Forward primer	GTTCACCTGGAGTGATGGTTCTC
		Reverse primer	AGGACATGCCAGGGTCACCTTT
Traf6	mouse	Forward primer	ATTTCATTGTCAACTGGGCA
		Reverse primer	TGAGTGTCCCATCTGCTTGA
Irak1	mouse	Forward primer	GAGACCCTTGCTGGTCAGAG
		Reverse primer	GCTACACCCACCCACAGAGT
Socs3	mouse	Forward primer	TCACTTTCTCATAGGAGTCCAGGT
		Reverse primer	CAAGAGAGCTTACTACATCTATTCTGG
β-actin	mouse	Forward primer	GTGCTATGTTGCTCTAGACTTCG
		Reverse primer	ATGCCACAGGATTCCATACC
miR146a-5p	mouse	–	TGAGAACTGAATTCCATGGGTT
miR221	mouse	–	ACCTGGCATACAATGTAGATTTCTGT
miR222	mouse	–	CTCAGTAGCCAGTGTAGATCC
U6	mouse	Forward primer	GGAACGATACAGAGAAGATTAGC
		Reverse primer	TGGAACGCTTCACGAATTTGCG

### 2.11 Flow Cytometry for macrophage subset analysis

PE-conjugated anti-F4/80 (BD Pharmingen, CA, USA, 565410) was used as a pan-macrophage cell surface marker. To further distinguish macrophage M1 and M2 polarization states, PE-Cy™7-conjugated anti-CD86 (BD Pharmingen, CA, USA, 560582) was chosen to mark the M1 phenotype, and APC-conjugated anti-CD206 (Invitrogen, CA, USA, 17-2061-82) was chosen to mark the M2 phenotype. M1 phenotype was labelled as F4/80^+^CD86^+^, whereas M2 phenotype was labelled as F4/80^+^CD206^+^ (30, 36).

RAW264.7 cells or macrophages isolated from the spleen of mice were suspended in cold stain buffer (FBS); this was followed by incubation with PE-conjugated anti-F4/80 and PE-Cy™7-conjugated anti-CD86 or APC-conjugated anti-CD206 antibodies on ice in a dark room for 30 min. Then we washed these cells three times with FBS and examined using a CytoFlex flow cytometer (Beckman Coulter, CA, USA) within 2 h. Flow cytometric images were analyzed using CytExpert software. Gating strategy is shown in the **Supplementary Figure S2**.

### 2.12 Immunoblotting

Proteins were harvested from cells *via* extraction with RIPA buffer. Proteins were separated with SDS-PAGE and then transferred onto PVDF membranes (Sigma, MO, USA), which were blocked with 5% skim milk for 1 h at 20–25°C and incubated overnight with the following antibodies at 4°C: TRAF6, dilution 1:1000 (Abcam, MA, USA, ab40675), p-P65, dilution 1:1000 (CST, MA, USA, #3033P); P65, dilution 1:1000 (CST, MA, USA, #8242S), p-IκBα, dilution 1:1000 (CST, MA, USA, #2859P), IκBα, dilution 1:1000 (CST, MA, USA, #4814P), p-IKK, dilution 1:1000 (CST, MA, USA, #2697P), IKK, dilution 1:1000 (CST, MA, USA, #2678P), and α-Tubulin, dilution 1:2000 (Proteintech, Wuhan, China, 11224-1-AP). The membranes were then incubated with HRP-conjugated goat anti-rabbit/mouse IgG (H+L) antibody (Antgene, Wuhan, China). Protein bands were visualized using ECL chemiluminescence substrates (Biosharp, Beijing, China, BL523B) and Image Lab 6.0 software (Bio-Rad, CA, USA) (37).

### 2.13 Fluorescent *in situ* Hybridization (FISH)

Biotin-labeled MIR222HG probes, FAM-labeled mir222hg, and Cy3-labeled miR146a-5p were designed and synthesized by GenePharma (Shanghai, China), and experiments were implemented according to the manufacturer’s instructions for the RNA Fish Kit (GenePharma, Shanghai, China) (38). Briefly, the cells were seeded, fixed with 4% paraformaldehyde, permeabilized, and prehybridized. The cells were then hybridized with RNA probe mix overnight. On the second day, cells were washed and stained with DAPI. Finally, images were observed and analyzed using a confocal microscope (Olympus FV1200, Tokyo, Japan). The sequences of MIR222HG, mir222hg, miR146a-5p, 18s and NC probes for FISH are listed in **Table 4**.

**Table 4:** List of probes used for FISH.

Gene symbol	Species	DNA(5'-3')	modifications	
			5'	3'
MIR222HG-1	Human	TACACAC+CCACAAA+GACAAAGG		3'Biotin
MIR222HG-2	Human	AAAGGA+TTTGCA+GAAACCCA+TG		3'Biotin
MIR222HG-3	Human	ACAA+GAGG+TTAGAGT+TGAGGAT		3'Biotin
MIR222HG-4	Human	ACACAA+TTGT+TCCA+GAACAGAC		3'Biotin
MIR222HG-5	Human	GCTG+GAAAAGA+TACTTCC+TGAA		3'Biotin
NC-Biotin	Human	TGCTT+TGCACGGTAACGCC+TGTTTT		3'Biotin
18s-Biotin	Human	CT+TCCT+TGGATGTGGT+AGCCGT+TTC		3'Biotin
miR146a-5p	Mouse	AACCCA+TGGA A+TTCAGTTC+T CA	5'CY3	
mir222hg-1	Mouse	AGTGCTGGAAAGGTACTAGG	5'FAM	
mir222hg-2	Mouse	TTCCTGGTGACAGAAAGCTG	5'FAM	
mir222hg-3	Mouse	CCAGGGAGAATAAAGGGCTG	5'FAM	
NC-CY3	Mouse	TGCTT+TGCACGGTAACGCC+TGTTTT	5'CY3	
NC-FAM	Mouse	TGCTT+TGCACGGTAACGCC+TGTTTT	5'FAM	
18s-FAM	Mouse	CTGCCTTCCTTGGATGTGGTAGCCGTTTC	5'FAM	

### 2.14 Dual-luciferase reporter assays

The targeted associations between mir222hg and miR146a-5p, and the association between miR146a-5p and Traf6, were made based on bioinformatic prediction and validated using dual luciferase reporter gene assays.

Wild type pmirGLO-mir222hg/Traf6 (WT) and mutant pmirGLO-mir222hg/Traf6-mut (MUT) reporter constructs were customed and synthesized by GenePharma (Shanghai, China). All plasmids were then co-transfected with miR146a-5p mimic or mimic NC in HEK293T cells for 24 h. We used a Dual Luciferase Reporter Assay Kit (Promega, Madison, WI, USA) to measure firefly luciferase levels and for the normalization of these levels to *Renilla* luciferase activity (38, 39). Each experiment was repeated at least three times.

### 2.15 Statistical analyses

All statistical analyses in our research were performed by using GraphPad Prism 6.0 (GraphPad Software, San Diego, CA, USA), and the values are presented as the mean ± SEMs. Statistical significance was assessed by Mann–Whitney U test or unpaired t-test for experiments comparing two groups appropriately, whereas two-way ANOVA followed by Sidak’s multiple comparisons test was used for comparisons between more than two groups, and the linear correlation between the transcript-levels of MIR222HG and macrophage inflammation-related genes involved in this study was drawn by using Spearman rank correlation. Differences were considered statistically significant at P < 0.05.

## 3 Results

### 3.1 Differentially expressed RNAs in allergic diseases and construction of a ceRNA network

**Figure 1:**
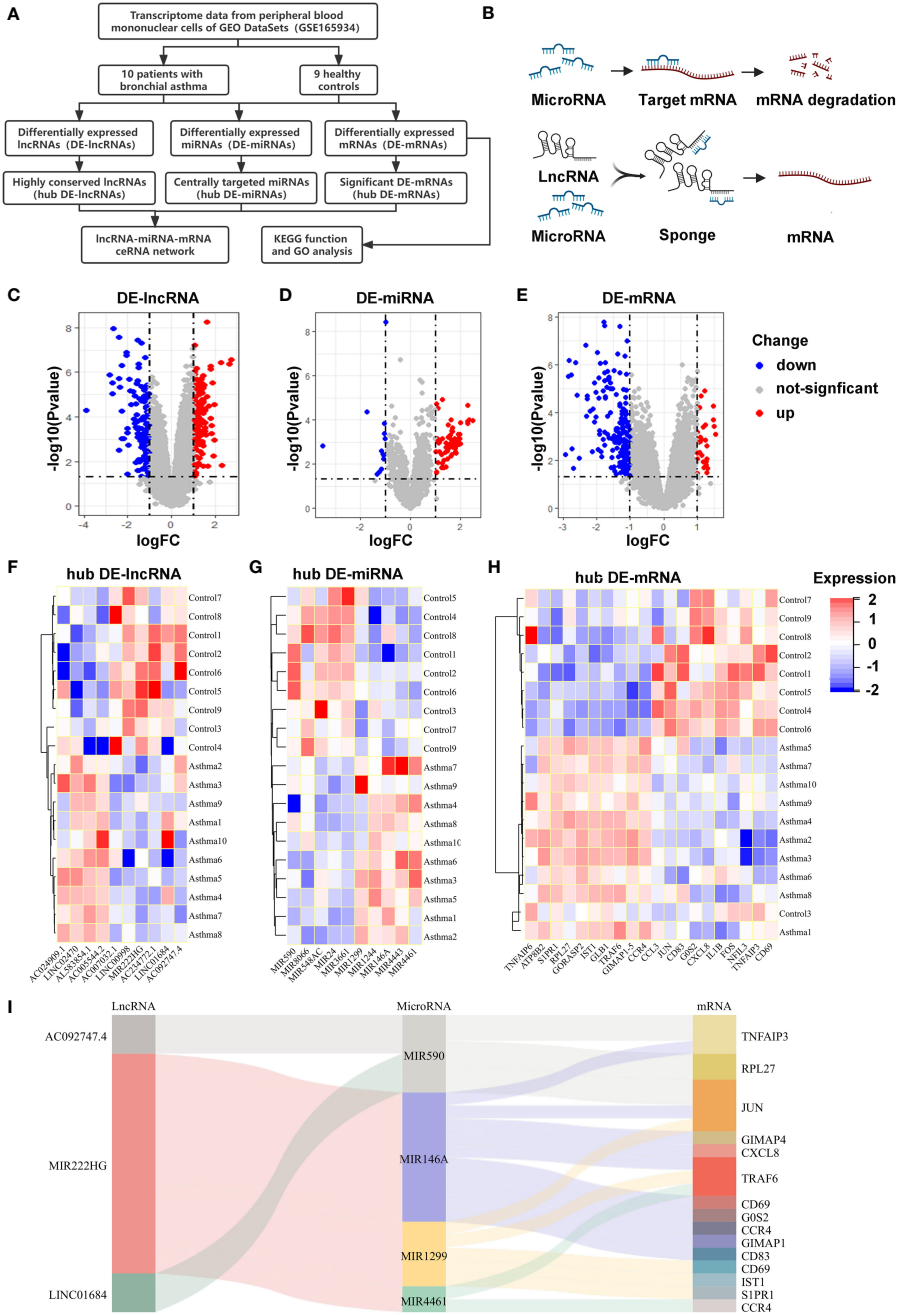
Differentially expressed RNAs in allergic diseases and construction of a ceRNA network **(A)** This flow chart showing the methodology of the bioinformatic analysis. **(B)** The interaction mechanism diagram of the ceRNA network. **(C-E)** Volcano plots of DE-lncRNAs **(C)**, DE-miRNAs **(D)** and DE-mRNAs **(E)** in PBMCs of allergic asthma, analyzed from the GEO database (GSE165934). **(F-H)** Heatmaps showing the selected hub DE-lncRNAs **(F)**, hub DE-miRNAs **(G)**, and hub DE-mRNAs **(H)**. **(I)** Sankey diagram showing the competing endogenous RNA network in allergic asthma. The screening criteria were |log_2_FC| ≥ 1 and P value < 0.05.

The ‘one airway, one disease’ theory suggests that asthma and AR could share common disease associations, immunopathological mechanisms, and genetic origin (40–42). Based on the theory, microarray data of allergic asthma (GSE165934) were downloaded and analyzed in the absence of AR-related microarray data in the GEO database. Subsequently, the DE-lncRNAs, DE-miRNAs, and DE-mRNAs were identified in patients with allergic asthma using the limma package. We identified 214 DE-lncRNAs (136 upregulated and 78 downregulated; **Figure 1C**), 90 DE-miRNAs (77 upregulated and 13 downregulated; **Figure 1D**), and 207 DE-mRNAs (31 upregulated and 176 downregulated; **Figure 1E**) from the GSE165934 dataset. Based on gene conservation and log_2_FC values, we selected 10 hub DE-lncRNAs, 10 hub DE-miRNAs, and 20 hub DE-mRNAs, to aid our ceRNA network construction. The distribution of DEGs was visualized using heatmaps (**Figures 1F–H**). Subsequently, to identify whether the hub DEGs existed in the competing endogenous regulatory network, the interactions of hub DE-lncRNAs–hub DE-miRNAs and hub DE-miRNAs–DE-mRNAs were predicted by bioinformatics analysis and integrated using the Cytoscape software. Finally, three hub DE-lncRNAs, four hub DE-miRNAs, and 15 DE-mRNAs were used to construct the ceRNA network, which was visualized using a Sankey diagram (**Figure 1I**). **Figures 1A, B** show the schemes for the workflow of our bioinformatics analyses and the interaction mechanism diagram of our ceRNA, respectively.

### 3.2 MIR222HG is significantly downregulated in allergic rhinitis

**Figure 2:**
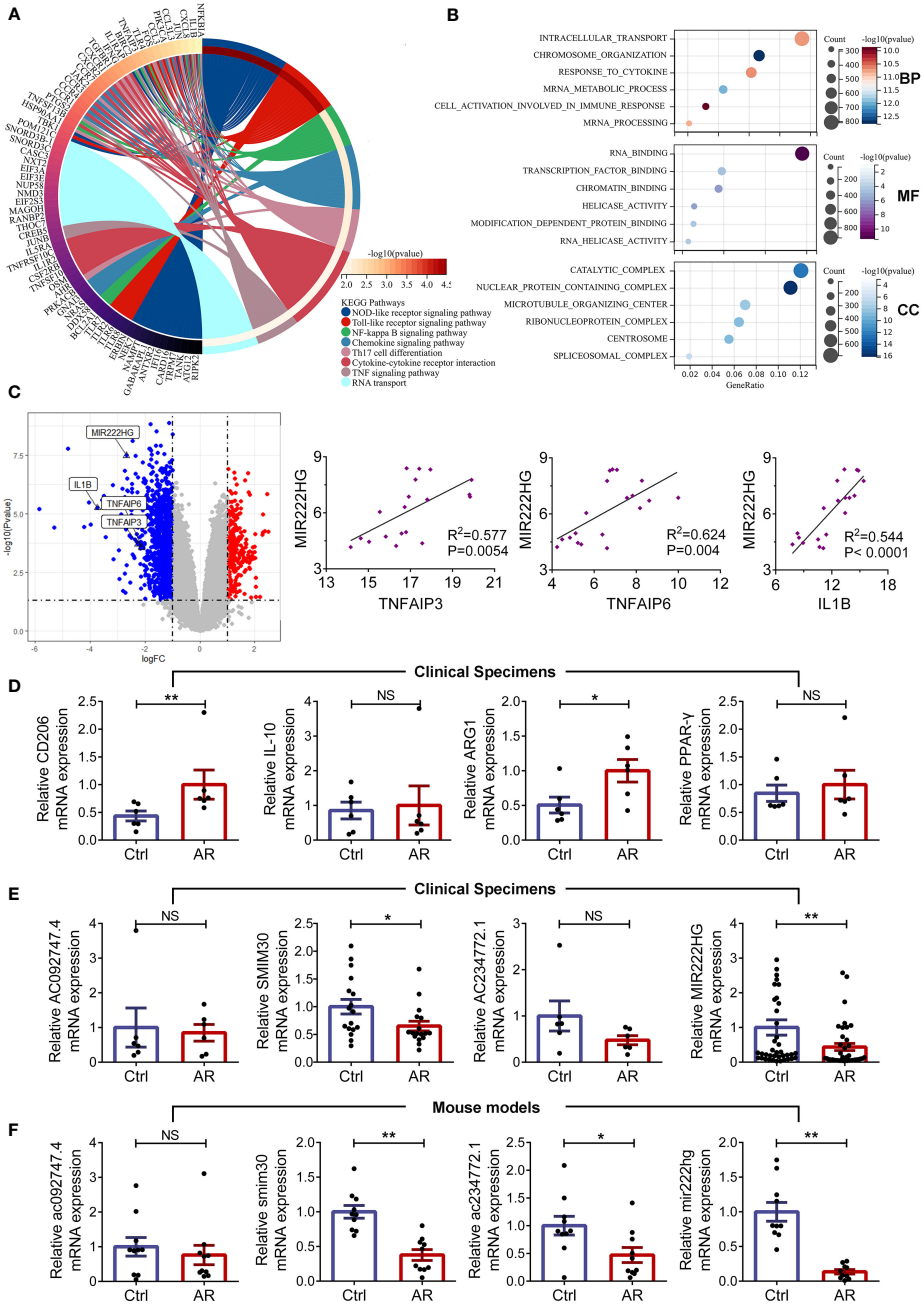
MIR222HG is significantly downregulated in allergic rhinitis **(A)** Circos plot displaying the Kyoto Encyclopedia of Genes and Genomes (KEGG) analyses of DE-mRNAs. **(B)** Bubble chart of Gene Ontology (GO) enrichment analyses of DE-mRNAs, including the biological process (BP), molecular function (MF) and cellular component (CC) categories. **(C)** Volcano plots highlighting the fold changes and significant differences in the expression of TNFAIP3, TNFAIP6, IL1B, and MIR222HG. Correlation analysis of the expression levels of TNFAIP3, TNFAIP6 or IL1B, and MIR222HG in the microarray data (Spearman rank correlation). **(D)** The relative mRNA level expression levels of CD206, IL-10, ARG1, and PPAR-γ were detected in AR patients and healthy controls by qRT-qPCR. **(E)** The relative transcript-level expression levels of AC092747.4, SMIM30, AC234772.1, and MIR222HG were validated in AR patients and healthy controls by qRT-qPCR. **(F)** The relative transcript-level expression levels of ac092747.4, smim30, ac234772.1, and mir222hg were validated in the animal model of AR. Each point represents data from one individual sample. Data are shown as the mean ± SEMs (n= at least 6 samples per group). Data are merged from three independent experiments. Statistical significance was calculated by unpaired t-test. * p< 0.05, ** p< 0.01, NS no significance.

To further investigate the roles of significantly dysregulated mRNAs in allergic diseases, we performed KEGG and GO enrichment analyses for DE mRNAs. Our enrichment analysis of the KEGG signaling pathway showed that DE-mRNAs not only were significantly clustered in the NOD-like receptor, the Toll-like receptor, NF-kB, chemokine and TNF signaling pathway, but also participated in Th17 cell differentiation, cytokine-cytokine receptor interaction, and RNA transport (**Figure 2A**). GO analysis of DE-mRNAs showed they were mainly enriched in mRNA metabolic processes, responses to cytokines, cell activation in immune responses, RNA binding, transcription factor binding, and chromatin binding pathways (**Figure 2B**). The results of the above KEGG and GO enrichment analyses indicate that dysregulated mRNAs in allergic diseases might be involved in macrophage-related inflammation pathways. Remarkably, our microarray analyses demonstrated MIR222HG was the most downregulated gene among the highly conserved hub DE-lncRNAs we had defined, and was positively correlated with macrophage-associated inflammatory molecules, such as TNFAIP3, TNFAIP6, and IL1B (**Figure 2C**). Immune cell composition analysis of microarray data showed that the abundance of M2 macrophages and activated mast cells were higher, while the abundance of naive CD4^+^T cells, resting CD4^+^ memory T cells and eosinophils were lower in PBMCs of patients with allergic asthma when compared with those in the controls (**Supplementary Figure S3A**). Then we performed matrix correlation analyses between the expression of four highly conserved hub DE-lncRNAs and the ratios of immune cells. The results showed that the transcript expression of AC092747.4 was correlated with the abundance of resting CD4 ^+^ memory T cells and eosinophils. The transcript expression of SMIM30 was inversely correlated with the abundance of M2 macrophages and activated mast cells. The transcript expression of AC234772.1 was correlated with the abundance in naive B cells and naive CD4^+^T cells. MIR222HG transcript expression was inversely correlated with M2 macrophages abundance (**Supplementary Figure S3B**).

Subsequently, we recruited 79 patients (39 cases of AR and 40 controls) and established animal models (10 AR mice and 10 controls). The detailed clinical characteristics of the enrolled patients are presented in the **Table 1**. HE and PAS staining demonstrated that the mice in the AR group exhibited epithelial ciliary loss, abundant inflammatory cells infiltrates, and goblet cell hyperplasia in the nasal mucosa, when compared with those in the controls (**Supplementary Figures S1A–C**). Furthermore, AR mice presented increased AR symptom scores, which indicated successful AR modeling (**Supplementary Figure S1D**). Subsequently, RNA was isolated from patient PBMCs and mice spleens, respectively. We found mRNA levels of the M2 macrophage markers (CD206 and ARG1) were elevated in patients with AR (**Figure 2D**). To validate the reliability of the GSE165934 dataset and refine candidate lncRNA inclusion, transcript-level expression of four highly conserved hub DE-lncRNAs was further validated by qRT-PCR, including AC092747.4 (murine ac092747.4), SMIM30 (murine smim30), AC234772.1 (murine ac234772.1), and MIR222HG (murine mir222hg). Similar to the microarray data results for PBMCs of patients with allergic asthma, the transcript-level expression of SMIM30 and MIR222HG was significantly decreased in PBMCs of AR patients when compared with those in the controls (**Figure 2E**). Furthermore, the expression levels of smim30, mir222hg, and ac234772.1 were significantly decreased in the spleen tissue of AR mice compared with those in the controls (**Figure 2F**). However, the transcript-level expression of AC092747.4, AC234772.1, and ac092747.4 did not show significant differences between the AR group and controls (**Figures 2E, F**).

Notably, among the four conserved hub DE-lncRNAs, we identified that the transcript-level expression of lncRNA-MIR222HG (mir222hg), which has an ambiguous annotated function *in vivo*, was robustly decreased in both patients with AR and AR mice. Surprisingly, MIR222HG was a pivotal component of our ceRNA network and was predicted to bind miR146a-5p, miR1299, and miR4461. Furthermore, based on its sequence, we predict miR146a-5p to bind to 3^’^ UTR of TRAF6, which is involved in macrophage inflammation and polarization pathways (43). We isolated macrophages from the spleen of AR mice for qRT-PCR and found that the transcript-level expression of mir222hg was decreased in macrophages isolated from the spleen of AR mice when compared with controls (**Supplementary Figure S3C**). After obtaining the results, we hypothesized that MIR222HG could act as a ceRNA sponge, which functions to adsorb miR146a-5p and upregulate TRAF6, thus regulating macrophage polarization, and consequently, AR pathogenesis.

### 3.3 Conservation analysis and subcellular localization of MIR222HG/mir222hg

**Figure 3:**
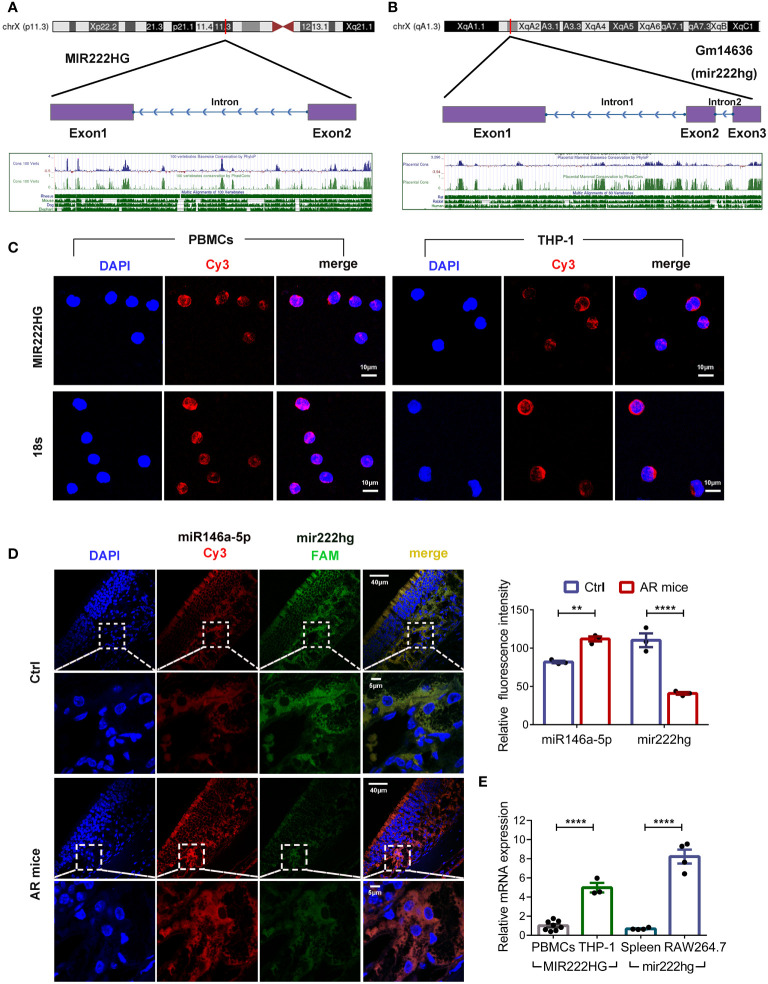
Conservation analysis and subcellular localization of MIR222HG/mir222hg **(A)** MIR222HG is mapped to the human chromosome X:45,745,211-45,770,274 with a high degree of conservation in its sequence. **(B)** Gm14636(mir222hg) is located on murine chromosome X:19,023,220-19,033,471 with a high degree of conservation in sequence. **(C)** Representative images of RNA-Fluorescence in Situ Hybridization (RNA-FISH) assay showing subcellular localization of MIR222HG in PBMCs and THP-1 cells (MIR222HG-Cy3-red; nucleus-DAPI-blue). 18s was used as the positive control. Scale bars, 10 μm. **(D)** Representative images (left) and quantification analysis (right) of RNA-FISH assay showing subcellular localization and expression of mir222hg and miR146a-5p in nasal mucosa tissues (miR146a-5p-Cy3-red; mir222hg-FAM-green; nucleus-DAPI-blue). Scale bars, 5 μm and 40 μm. **(E)** The qRT-qPCR was used to detect the difference in relative transcript-level expression of MIR222HG/mir222hg between human PBMCs, THP-1 cells, mouse spleen cells, and RAW264.7 cells. Each point represents data from one individual sample. Data are shown as the mean ± SEMs (n= at least 3 samples per group). Data are merged from three independent experiments. **(C, D)** Images are representative of three independent experiments. Statistical significance was assessed by unpaired t-test for experiments comparing two groups, whereas two-way ANOVA followed by Sidak’s multiple comparisons test was used for comparisons between more than two groups. ** p< 0.01, **** p< 0.0001.

Bioinformatic analyses showed that MIR222HG was located on chromosome X:45,745,211-45,770,274 (UCSC-Human GRCh38/hg38) with two exons (**Figure 3A**). However, lncRNAs have inferior species conservation properties, and the regulation and function of MIR222HG remains unclear, as few articles in the literature have focused on it. To assess whether our constructed ceRNA network for PBMCs from patients with allergic asthma could apply to different species, we analyzed the sequence conservation properties of MIR222HG using NONCODE BLAST. Surprisingly, we found that the ENSMUST00000143129.2 transcript of the Gm14636 gene located on chromosome X:19,023,220-19,033,471 (UCSC-Mouse GRCm39/mm39) matched with MIR222HG and was therefore designated as mir222hg for convenience (**Figure 3B**). Coincidentally, a competing endogenous regulatory network has also been predicted in murine mir222hg/miR146a-5p/Traf6.

The biological functions of lncRNAs depend largely on their unique intracellular localization. Subcellular localization of MIR222HG/mir222hg was investigated in human PBMCs, THP-1 cells, and mouse nasal mucosa tissues using a FISH assay. The results showed that MIR222HG was mainly expressed in the cytoplasm of the human PBMCs and THP-1 cells (**Figure 3C**). FISH assay results of mouse nasal mucosa tissues showed that mir222hg was also localized to the cytoplasm (**Figure 3D**). Furthermore, compared with the controls, the AR group exhibited significantly reduced mir222hg levels, concomitant with significantly increased miR146a-5p levels in nasal mucosa tissues (**Figure 3D**). Furthermore, FISH demonstrated the colocalization of mir222hg and miR146a-5p in nasal mucosa tissues (**Figure 3D**). To test the hypothesis of ceRNA above in macrophages, we compared MIR222HG/mir222hg expression among human PBMCs, THP-1 cells, mouse spleen cells, and RAW264.7 cells. Relative to in human PBMCs and mouse spleen cells, MIR222HG/mir222hg was over expressed in monocyte-macrophage cell lines, such as RAW264.7 cells and THP-1 cells (**Figure 3E**).

### 3.4 Mir222hg is significantly upregulated in LPS-stimulated M1 macrophage and downregulated in IL-4/ovalbumin-stimulated M2 macrophage

**Figure 4:**
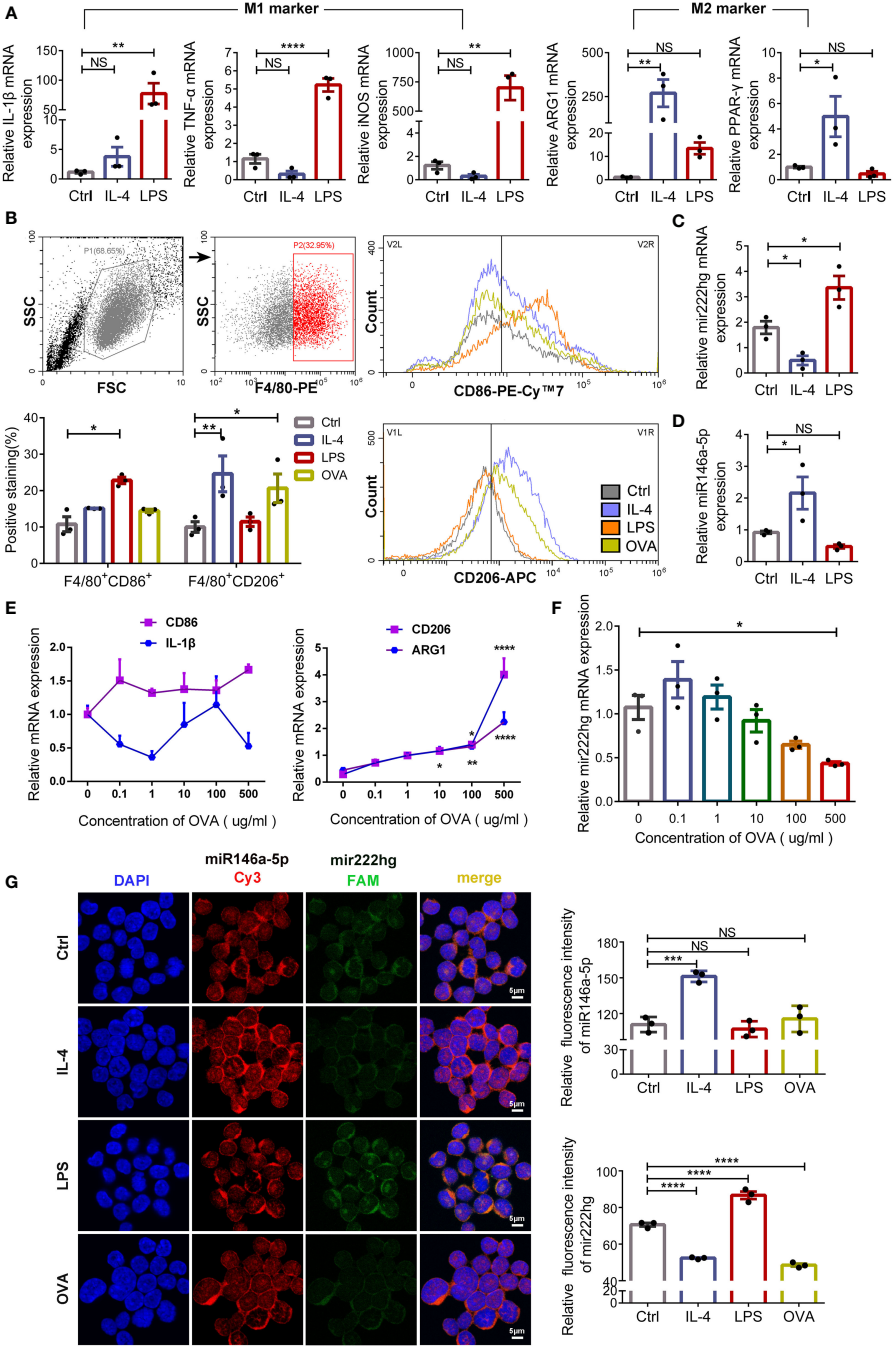
Mir222hg is significantly upregulated in LPS-stimulated M1 macrophage and downregulated in IL-4/ovalbumin-stimulated M2 macrophage **(A)** The qRT-qPCR showing IL-1β, TNF-α, iNOS, ARG1, and PPAR-γ relative mRNA expression in LPS-induced M1 and IL-4-induced M2 macrophages. **(B)** Representative flow cytometry plots and statistical analysis showing the surface expression of CD86 and CD206 in LPS-induced M1 and IL-4/OVA-induced M2 macrophages. **(C, D)** The relative transcript-level expression of mir222hg **(C)** and miR146a-5p **(D)** in LPS-induced M1 and IL-4-induced M2 macrophages were analyzed by qRT-PCR. **(E, F)** The qRT-qPCR showing CD86, IL-1β, CD206, ARG1, and mir222hg relative mRNA expression in RAW264.7 cells treated with different concentrations of OVA (0.1-500 μg/ml) for 48 h. β-actin was used as an internal control. **(G)** Representative images (left) and quantification analysis (right) of RNA-FISH assay showing subcellular localization and expression of mir222hg and miR146a-5p in RAW264.7 cells treated with LPS, IL-4 and OVA, respectively (miR146a-5p-Cy3-red; mir222hg-FAM-green; nucleus-DAPI-blue). Scale bars, 5 μm. Each point represents data from one individual sample. Data are shown as the mean ± SEMs (n= at least 3 samples per group). Data are merged from three independent experiments. Statistical significance was calculated by two-way ANOVA followed by Sidak’s multiple comparisons test. * p< 0.05, ** p< 0.01, *** p< 0.001, **** p< 0.0001, NS no significance.

To test the role of the mir222hg/miR146a-5p/Traf6 axis in macrophage polarization, RAW264.7 cells were induced to the M1 macrophage following treatment with LPS for 24 h and M2 macrophage following treatment with IL-4 for 48 h. Subsequently, qRT-PCR was performed to confirm whether the polarization of M1 and M2 phenotype macrophages was successfully induced. qRT-PCR revealed that, compared with the untreated control, the M1 macrophage markers IL-1β, TNF-α, and iNOS were upregulated in LPS-treated macrophages (**Figure 4A**). Moreover, the M2 macrophage markers ARG1 and PPAR-γ were upregulated significantly in the IL-4-treated macrophages when compared with those in the control (**Figure 4A**). Flow cytometry was used to determine the levels of M1 surface marker CD86 and M2 surface marker CD206. The levels of CD86 were elevated in LPS-treated macrophages when compared with those in the control. Meanwhile, CD206 expression was significantly upregulated in IL-4-treated macrophages (**Figure 4B**). The findings suggest that the polarization of M1 and M2 macrophages was induced successfully.

Furthermore, mir222hg was significantly upregulated in LPS-treated M1 macrophages and downregulated in IL-4-treated M2 macrophages (**Figure 4C**). The transcript-level expression of miR146a-5p was increased in IL-4-treated M2 macrophages (**Figure 4D**), but the transcript-level expression of miR221/222 was not affected by the polarization of macrophages (**Supplementary Figure S3D**). The results suggest that mir222hg and miR146a-5p may be involved in macrophage polarization regulation, which is unrelated to miR221/222.

To induce allergy at the cellular level, RAW264.7 cells were further treated with various concentrations of OVA (0.1-500 μg/mL) for 48 h to evaluate the effect of OVA on macrophage polarization. We found that the mRNA expression of M2 macrophage markers CD206 and ARG1 was upregulated with an increase in OVA concentrations used to stimulate RAW264.7 cells. However, no significant change was detected in the mRNA expression of M1 macrophage markers CD86 and IL-1β (**Figure 4E**). The result indicates that a higher concentration of OVA facilitated the polarization of macrophages to the M2 phenotype.

Meanwhile, the transcript-level expression of mir222hg decreased with an increase in OVA concentration (**Figure 4F**). To further evaluate the transcript-level expression and localization of mir222hg and miR146a-5p, FISH was performed. We found that mir222hg was downregulated in IL-4-treated M2 macrophages and OVA-treated macrophages (**Figure 4G**). Simultaneously, miR146a-5p was upregulated in IL-4-treated M2 macrophages and co-localized with mir222hg in RAW264.7 cells (**Figure 4G**).

### 3.5 Overexpression or knockdown of mir222hg correlate with macrophage polarization

**Figure 5:**
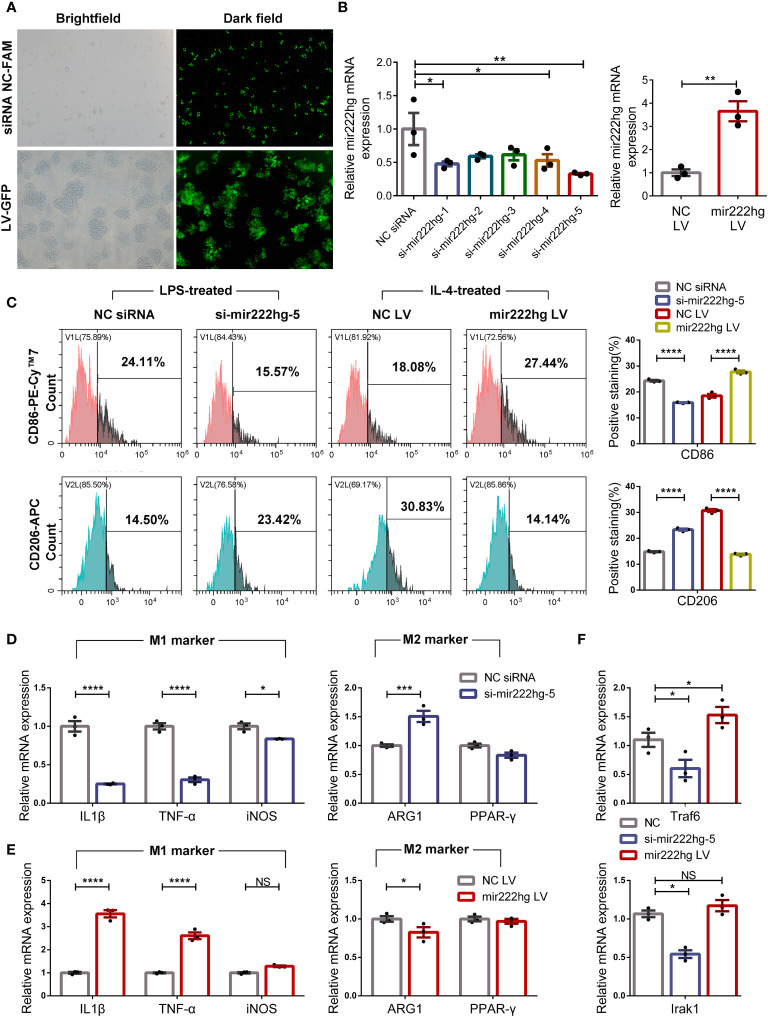
Overexpression or knockdown of mir222hg correlated with macrophage polarization RAW264.7 cells were transfected with si-mir222hg-1-5, NC siRNA, mir222hg LV, or NC LV. Cells transfected with si-mir222hg-5 and NC siRNA were treated with 100 ng/ml LPS for 24 h, whereas those transfected with mir222hg LV and NC LV were treated with 40 ng/ml IL-4 for 48 h. **(A, B)** Immunofluorescence analysis **(A)** and qRT-PCR **(B)** assays were performed to verify the transfection efficiency of the siRNA and overexpressing lentivirus. **(C)** Representative flow cytometry histograms (left) and statistical analysis (right) showing the surface expression of CD86 and CD206. **(D-F)** qRT-PCR showing IL-1β, TNF-α, iNOS, ARG1, PPAR-γ, Traf6, and Irak1 relative mRNA expression. Each point represents data from one individual sample. Data are shown as the mean ± SEMs (n= 3 per group). Data are merged from three independent experiments. Statistical significance was assessed by unpaired t-test for experiments comparing two groups, whereas two-way ANOVA followed by Sidak’s multiple comparisons test was used for comparisons between more than two groups. * p< 0.05, ** p< 0.01, *** p< 0.001, **** p< 0.0001, NS no significance.

To explore the association between mir222hg and macrophage polarization, we conducted gain-and loss-of-function assays of mir222hg in RAW264.7 cells by transfection with mir222hg LV and si-mir222hg-1–5 respectively. Immunofluorescence and qRT-PCR were performed to verify the transfection efficiency. As shown in **Figure 5A**, the transfection efficiency of mir222hg LV and si-mir222hg-1–5 was > 80%. Moreover, qRT-PCR demonstrated that ectopic expression of mir222hg increased the transcript-levels of mir222hg significantly, whereas mir222hg knockdown (si-mir222hg-5) resulted in approximately a 60% reduction in mir222hg expression in RAW264.7 cells (**Figure 5B**).

Because mir222hg was downregulated in IL-4-treated M2 macrophages and upregulated in LPS-treated M1 macrophages, RAW264.7 cells transfected with si-mir222hg-5 were treated with LPS, and RAW264.7 cells transfected with mir222hg LV were stimulated with IL-4. The expression of CD86 decreased and that of CD206 increased in LPS-treated macrophages transfected with si-mir222hg-5 when compared with the NC siRNA group. Conversely, the overexpression of mir222hg increased the expression of CD86 and decreased the expression of CD206 in IL-4-treated macrophages (**Figure 5C**).

qRT-PCR demonstrated that the mRNA expression of M1 markers decreased, whereas that of M2 marker ARG1 increased, in LPS-treated macrophages transfected with si-mir222hg-5 (**Figure 5D**). Conversely, the overexpression of mir222hg increased M1 marker mRNA expression and decreased ARG1 mRNA expression in IL-4-treated macrophages (**Figure 5E**). Moreover, in cultured RAW264.7 cells without any treatment, the mRNA expression of Traf6 and Irak1 was decreased in the si-mir222hg-5 group and increased in the mir222hg LV group (**Figure 5F**). The data indicate that mir222hg potentially represses M2 macrophage polarization.

### 3.6 MiR146a-5p knockdown facilitates macrophage M1 polarization and reverses M2 polarization caused by silencing mir222hg

**Figure 6:**
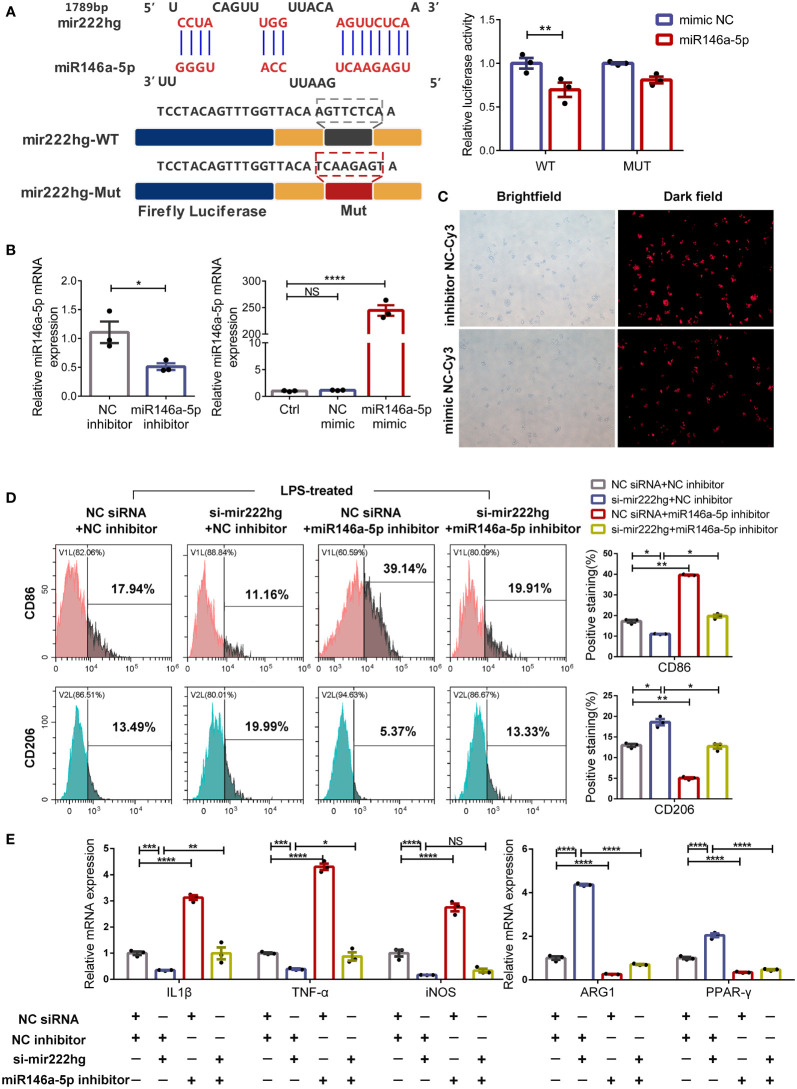
MiR146a-5p knockdown facilitates macrophage M1 polarization and reverses M2 polarization caused by silencing mir222hg **(A)** A schematic to show the putative mir222hg binding sites in miR146a-5p. The sequences of mir222hg-WT and mir222hg-Mut are as listed. Luciferase reporter gene assays were performed to validate the correlation between mir222hg and miR146a-5p in 293T cells. **(B, C)** RAW264.7 cells were transfected with NC siRNA + NC inhibitor, si-mir222hg + NC inhibitor, NC siRNA + miR146a-5p inhibitor, or si-mir222hg + miR146a-5p inhibitor, respectively. The above transfected RAW264.7 cells were then treated with 100 ng/ml LPS for 24 h. qRT-PCR assay **(B)** and immunofluorescence analysis **(C)** were performed to verify the transfection efficiency. **(D)** Representative flow cytometry histograms (left) and statistical analysis (right) showing the surface expression of CD86 and CD206. **(E)** The qRT-qPCR showing IL-1β, TNF-α, iNOS, ARG1, and PPAR-γ relative mRNA expression. Each point represents data from one individual sample. Data are shown as the mean ± SEMs (n= 3 per group). Data are merged from three independent experiments. Statistical significance was assessed by unpaired t-test for experiments comparing two groups, whereas two-way ANOVA followed by Sidak’s multiple comparisons test was used for comparisons between more than two groups. * p< 0.05, ** p< 0.01, *** p< 0.001, **** p< 0.0001, NS no significance.

Bioinformatics analyses revealed that miR146a-5p contained one conserved target site of mir222hg. We therefore co-transfected mir222hg-WT/MUT plasmids with mimic miR146a-5p or mimic NC into 293T cells and conducted a luciferase reporter assay to study the interaction correlation between mir222hg and miR146a-5p. Mimic miR146a-5p decreased the luciferase activity of mir222hg compared to the mimic NC group. Conversely, transfection of mir222hg-MUT together with the mimic miR146a-5p did not influence the luciferase activity significantly (**Figure 6A**). The findings suggest that the correlation between mir222hg and miR146a-5p was mediated by the putative binding site.

To further explore whether mir222hg regulates macrophage polarization by sponging miR146a-5p, rescue experiments were carried out using an miR146a-5p inhibitor and miR146a-5p mimic. As shown in **Figure 6C**, the transfection efficiency of the miR146a-5p inhibitor and miR146a-5p mimic was above 80%. Furthermore, the miR146a-5p inhibitor resulted in around a 50% reduction in miR146a-5p transcript-level expression, whereas the miR146a-5p mimic significantly increased the levels of miR146a-5p transcript (**Figure 6B**).

Subsequently, we transfected the RAW264.7 cells with NC siRNA + NC inhibitor, si-mir222hg + NC inhibitor, NC siRNA + miR146a-5p inhibitor, or si-mir222hg + miR146a-5p inhibitor, and then stimulated the cells with LPS. As expected, flow cytometry analysis revealed that CD86 expression was downregulated and the expression of CD206 increased in LPS-treated macrophages transfected with si-mir222hg-5. However, the effects of si-mir222hg on marker expression were blocked in the si-mir222hg + miR146a-5p inhibitor group (**Figure 6D**). In addition, qRT-PCR results demonstrated that si-mir222hg + NC inhibitor decreased M1 marker mRNA expression and increased M2 marker mRNA expression in LPS-treated macrophages, compared with the NC siRNA + NC inhibitor group, whereas the miR146a-5p inhibitor blocked the effects of si-mir222hg on the mRNA expression of the markers (**Figure 6E**; **Supplementary Figure S4D**). Absolute quantification showed that there were 24–94 mir222hg molecules per cell versus 92–272 miR146a-5p molecules (**Supplementary Figures S4A–C**). Although the absolute copy number of mir222hg was slightly lower than that of miR146a-5p, miR146a-5p was not highly abundant in RAW264.7 cells, and it had a high affinity to mir222hg (8mer site). This indicates that mir222hg has the potential to act as a ceRNA (19). Overall, the results suggested that mir222hg could mediate macrophage polarization by sponging miR146a-5p.

### 3.7 MiR146a-5p overexpression facilitates macrophage M2 polarization and reverses M1 polarization caused by mir222hg overexpression

Further rescue experiments were carried out to test the roles of mir222hg and miR146a-5p in the regulation of macrophage polarization and AR pathogenesis. We transfected negative control lentivirus (NC LV) + NC mimic, mir222hg LV + NC mimic, NC LV + miR146a-5p mimic, or mir222hg LV + miR146a-5p mimic into RAW264.7, and then treated the cells with IL-4 and OVA, respectively.

**Figure 7:**
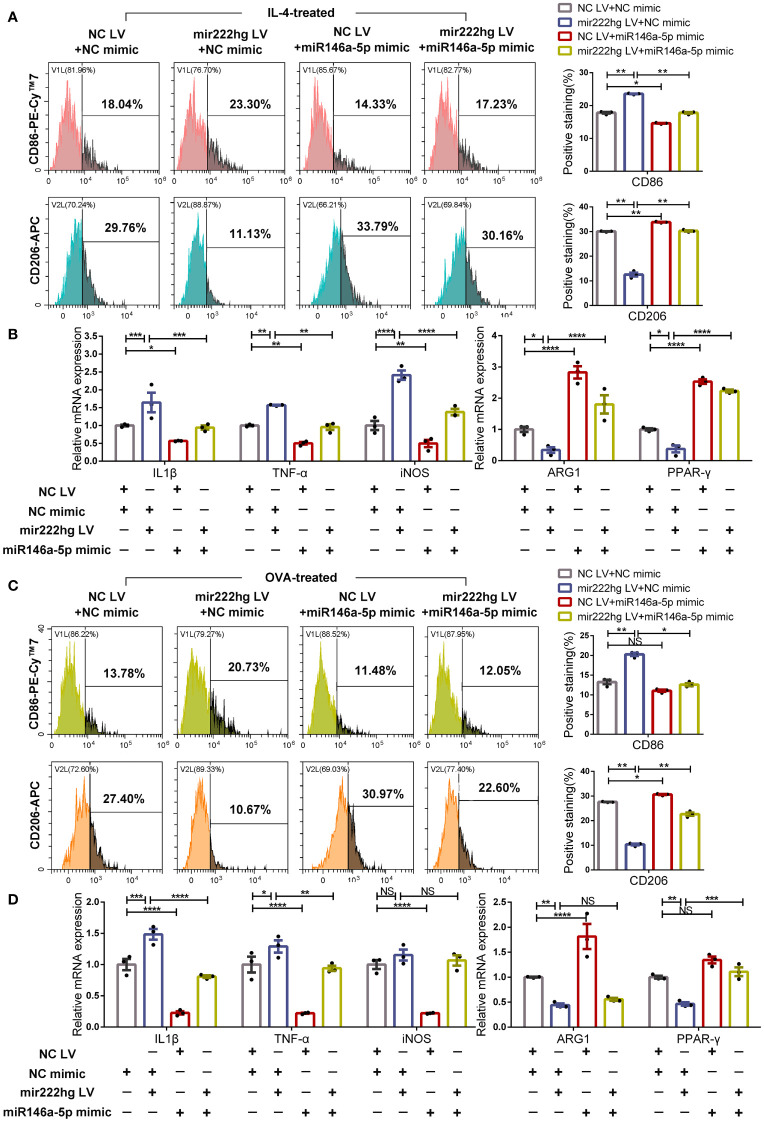
MiR146a-5p overexpression facilitates macrophage M2 polarization and reverses M1 polarization caused by mir222hg overexpression RAW264.7 cells were transfected with NC LV + NC mimic, mir222hg LV + NC mimic, NC LV + miR146a-5p mimic, or mir222hg LV + miR146a-5p mimic. **(A, B)** The transfected RAW264.7 cells were treated with 40 ng/ml IL-4 for 48 h. **(A)** Representative flow cytometry histograms (left) and statistical analysis (right) showing the surface expression of CD86 and CD206. **(B)** qRT-PCR was performed to detect IL-1β, TNF-α, iNOS, ARG1, and PPAR-γ relative mRNA expression. **(C, D)** The transfected RAW264.7 cells were treated with 500 μg/ml OVA for 48 h. **(C)** Representative flow cytometry histograms (left) and statistical analysis (right) showing the surface expression of CD86 and CD206. **(D)** qRT-PCR was conducted to detect IL-1β, TNF-α, iNOS, ARG1, and PPAR-γ relative mRNA expression. Each point represents data from one individual sample. Data are shown as the mean ± SEMs (n= 3 per group). Data are merged from three independent experiments. Statistical significance was calculated by two-way ANOVA followed by Sidak’s multiple comparisons test. * p< 0.05, ** p< 0.01, *** p< 0.001, **** p< 0.0001, NS no significance.

Flow cytometry demonstrated that the expression of CD86 increased and the expression of CD206 decreased in mir222hg LV + NC mimic-transfected macrophages following IL-4 stimulation, when compared with the NC LV + NC mimic. However, the miR146a-5p mimic promoted macrophage M2 polarization and reversed the M1 macrophage polarization caused by mir222hg LV (**Figure 7A**).

Consistently, qRT-PCR showed that mir222hg LV resulted in increased mRNA levels of the M1 markers IL-1β, TNF-α, and iNOS, and reduced those of the M2 markers ARG1, PPAR-γ, in IL-4 stimulated macrophages. Meanwhile the miR146a-5p mimic reversed the promotion of M1 macrophage polarization caused by mir222hg LV (**Figure 7B**; **Supplementary Figure S4E**). The above results indicate that the inhibition of M2 macrophage polarization by mir222hg LV in IL-4-treated macrophages could be reversed by the miR146a-5p mimic.

Furthermore, we stimulated the transfected cells with OVA and simulated allergy at the cellular level. The flow cytometry suggested that the expression of CD86 increased and the expression of CD206 decreased in mir222hg LV + NC mimic-transfected macrophages with OVA stimulation compared to the NC LV + NC mimic. The effects of mir222hg LV on marker expression were inhibited in the mir222hg LV + miR146a-5p mimic group (**Figure 7C**).

Upregulation of Traf6 and several M1 markers (IL-1β, TNF-α, and iNOS) and downregulation of the M2 markers (ARG1, PPAR-γ, IL-10 and YM1) caused by mir222hg LV in OVA-stimulated macrophages was abrogated by the miR146a-5p mimic (**Figure 7D**; **Supplementary Figure S4F**). The findings suggest that the inhibition of OVA-treated M2 macrophage polarization by mir222hg LV could be reversed by the miR146a-5p mimic.

Overall, whether the M2 macrophage polarization was activated by IL-4 or OVA, miR146a-5p overexpression promoted M2 macrophage polarization and reversed M2 macrophage weakness caused by overexpressing mir222hg.

### 3.8 Mir222hg/miR146a-5p/Traf6 axis modulates macrophage polarization *via* IKK/IκB/NF-кB signaling pathway

**Figure 8:**
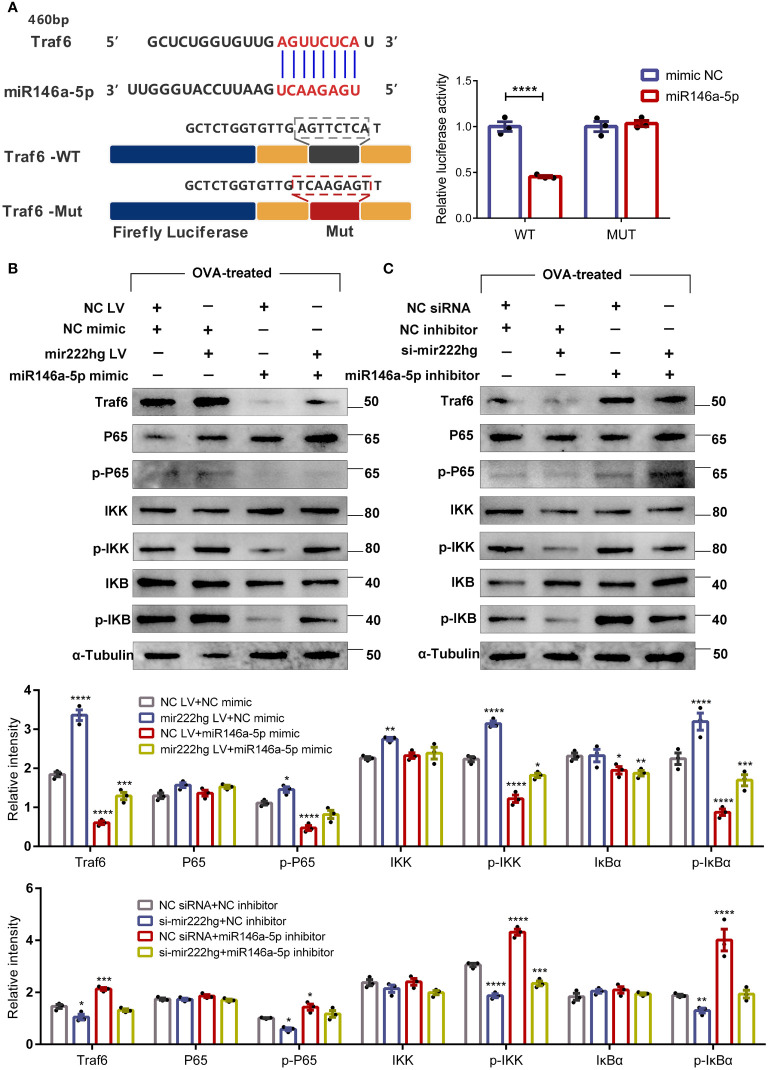
Mir222hg/miR146a-5p/Traf6 axis modulated macrophage polarization *via* the IKK/IκB/NF-кB signaling pathway **(A)** A schematic showing the putative miR146a-5p binding sites with the 3’-UTR of Traf6. The sequences of Traf6-WT and Traf6-Mut are as listed. Luciferase reporter gene assays were performed to validate the correlation between Traf6 and miR146a-5p in 293T cells. **(B, C)** RAW264.7 cells were transfected with NC siRNA + NC inhibitor, si-mir222hg + NC inhibitor, NC siRNA + miR146a-5p inhibitor, si-mir222hg + miR146a-5p inhibitor, NC LV + NC mimic, mir222hg LV + NC mimic, NC LV + miR146a-5p mimic, or mir222hg LV + miR146a-5p mimic, respectively. The transfected RAW264.7 cells were then treated with 500 μg/ml OVA for 48 h. The relative protein expression levels of Traf6, P65, p-P65, IκBα, p-IκBα, IKK, and p-IKK were determined by western blotting in transfected cells after normalization to α-Tubulin. Each point represents data from one individual sample. Data are shown as the mean ± SEMs (n= 3 per group). Data are merged from three independent experiments. Blots are representative of three independent experiments. Statistical significance was assessed by unpaired t-test for experiments comparing two groups, whereas two-way ANOVA followed by Sidak’s multiple comparisons test was used for comparisons between more than two groups. * p< 0.05, ** p< 0.01, *** p< 0.001, **** p< 0.0001.

Bioinformatics analyses showed that there were common miRNA response elements (MRE) with the sequence AGUUCUCA, between mir222hg and Traf6, which were predicted to bind to the sequence UCAAGAGU of miR146a-5p. Therefore, the association between miR146a-5p and Traf6 was validated using a dual luciferase reporter gene assay. When the binding motifs on miR146a-5p at 460 bp, 524 bp, 1366 bp, and 3860 bp of Traf6 were mutated to Traf6-MUT, luciferase activity was unaffected, compared with its decreasing activity in the presence of Traf6-WT (**Figure 8A**). This further validated the complementary binding of miR146a-5p to the 3’-UTR of Traf6.

MiR146a-5p, an NF-κB-dependent miRNA, downregulates NF-κB pathway-associated proteins. Therefore, we assessed the effect of mir222hg and miR146a-5p on the expression of Traf6, p-P65, p-IκBα, and p-IKK by immunoblotting. RAW264.7 cells were transfected and then treated with 500 μg/ml OVA for 48 h. The results suggested that the si-NC + miR146a-5p inhibitor increased the expression of Traf6, p-P65, p-IκBα, and p-IKK in transfected macrophages compared to the NC siRNA + NC inhibitor group, whereas the si-mir222hg blocked the effects of miR146a-5p inhibitor on the IKK/IκB/P65 signaling pathway (**Figure 8B**). The expression of Traf6, p-P65, p-IκBα, and p-IKK decreased in NC LV + miR146a-5p mimic-transfected macrophages when compared with the NC LV + NC mimic. The effects of the miR146a-5p mimic on the IKK/IκB/P65 signaling pathway were inhibited in the mir222hg LV + miR146a-5p mimic group (**Figure 8C**).

Cumulatively, the results strongly suggest that lncRNA-MIR222HG can act as a ceRNA sponge, competitively adsorb miR146a-5p, and activate the TRAF6/IKK/IκB/P65 signaling pathway, thus regulating macrophage polarization, and consequently, AR pathogenesis.

### 3.9 Intranasal administration of mir222hg–overexpressing lentivirus alleviated the allergic inflammatory response in AR mice

**Figure 9:**
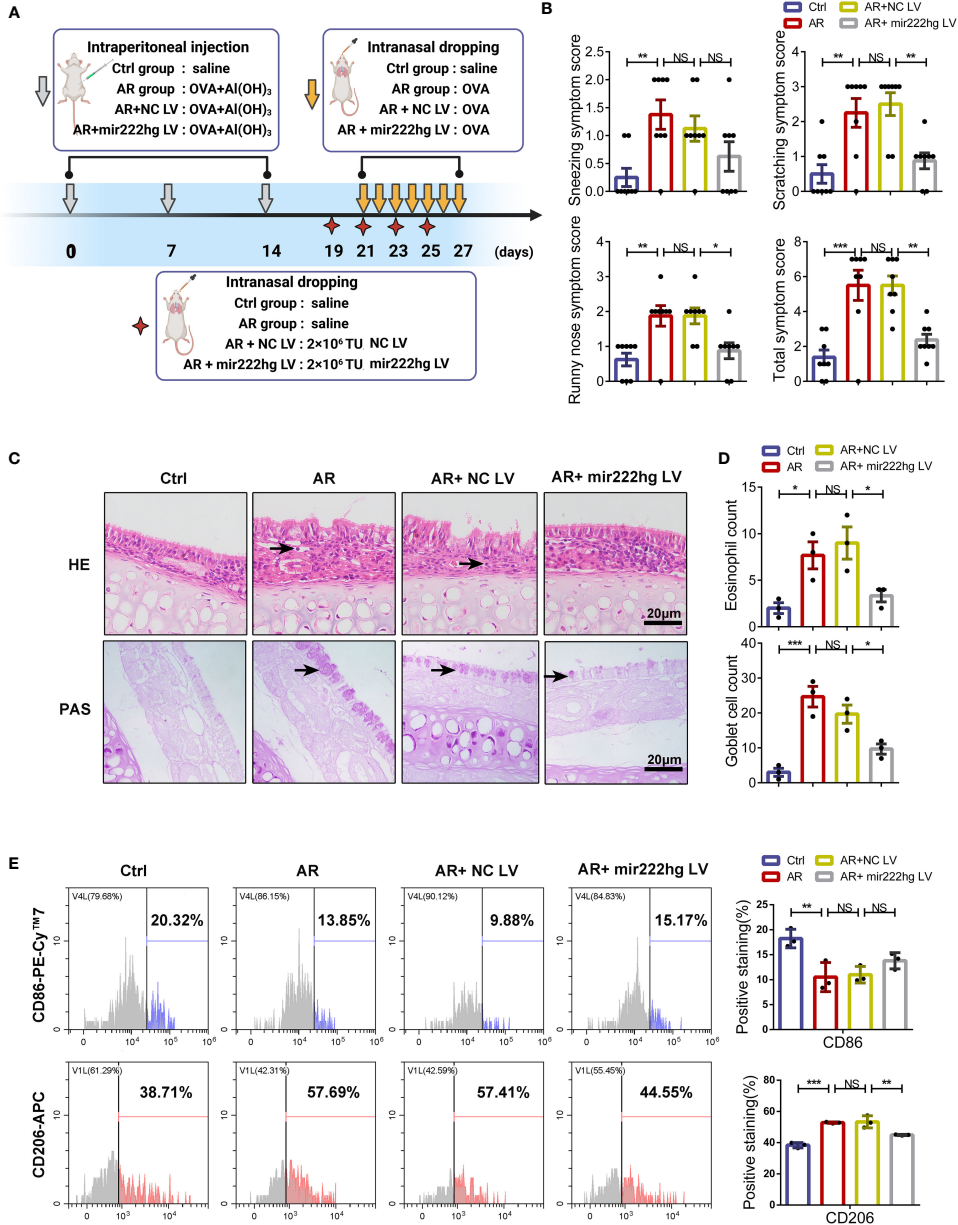
Intranasal administration of mir222hg–overexpressing lentivirus alleviated the allergic inflammatory response in AR mice **(A)** A schematic illustrating the experimental protocol for the establishment of the AR animal model and the intranasal administration of mir222hg–overexpressing lentivirus. **(B)** The partial and total AR nasal symptom scores of mice in the Ctrl, AR, AR + NC LV, and AR + mir222hg LV groups. **(C)** Representative images of nasal mucosa stained with HE and PAS. Eosinophils and goblet cells were labeled by arrows. Scale bars, 20 μm. **(D)** The eosinophil and goblet cell count in the nasal mucosa of mice in each group. **(E)** Representative flow cytometry histograms (left) and statistical analysis (right) showing the surface expression of CD86 and CD206 in macrophages isolated from the spleens of mice in each group. Each point represents data from one individual sample. Data are shown as the mean ± SEMs (n= at least 3 samples per group). Data are merged from three independent experiments. Statistical significance was calculated by two-way ANOVA followed by Sidak’s multiple comparisons test. * p< 0.05, ** p< 0.01, *** p< 0.001, NS no significance.

To assess the role of mir222hg in the regulation of allergic inflammation *in vivo*, an AR mouse model was established and intranasally administered with mir222hg-overexpressing or negative control lentivirus. The experimental protocols for the establishment of the AR animal model and the intranasal administration of lentivirus are shown in the schematic diagram (**Figure 9A**). Compared to the control group, mice in the AR group demonstrated increased allergic symptom scores, whereas there was no significant difference between the AR group and the AR + NC LV group. Compared with the AR + NC LV group, mice in the AR + mir222hg LV group exhibited less nasal scratching and nose running (**Figure 9B**). AR pathologies developed in the AR group and the AR + NC LV group, as indicated by the significant infiltration of nasal mucosa tissue by inflammatory cells, such as eosinophils and mast cells, along with the occurrence of goblet cell hyperplasia and nasal epithelial cilia destruction. None of this was observed in the control group. Notably, mice in the AR + mir222hg LV group displayed decreased eosinophil infiltration, fewer goblet cells, hyperplasia, and alleviative nasal epithelial cilia destruction in nasal mucosa tissue (**Figures 9C, D**).

To further verify whether mir222hg alleviated allergic inflammation through the regulation of macrophage polarization *in vivo*, we compared the surface expression of CD86 and CD206 in macrophages isolated from the spleens of different groups of mice. The flow cytometry suggested that the expression of CD86 decreased and the expression of CD206 increased in the AR and AR + NC LV groups when compared with the control group. The effects of allergens on macrophage polarization were alleviated in the AR + mir222hg LV group (**Figure 9E**). Consistently, qRT-PCR showed that mir222hg LV resulted in increased mRNA levels of Traf6 and iNOS, and reduced those of ARG1 in AR mice (**Supplementary Figures S5A, B**).

Mir222hg is the host gene of miR221/222, which might inhibit the Socs3/Traf6/NF-кB signaling pathway (44, 45) and modulate inflammatory Th17 cell response (46, 47). To exclude the impact of miR221/222, we detected the expression levels of miR221/222 and its downstream Socs3 in the spleen tissue of different groups of mice. qRT-PCR demonstrated that the mRNA expression of miR221/222 and its downstream Socs3 did not show significant differences between groups (**Supplementary Figures S5C, D**). The results suggest that intranasal administration of mir222hg–LV attenuated M2 macrophage polarization in AR in an miR221/222 independent manner.

**Figure 10:**
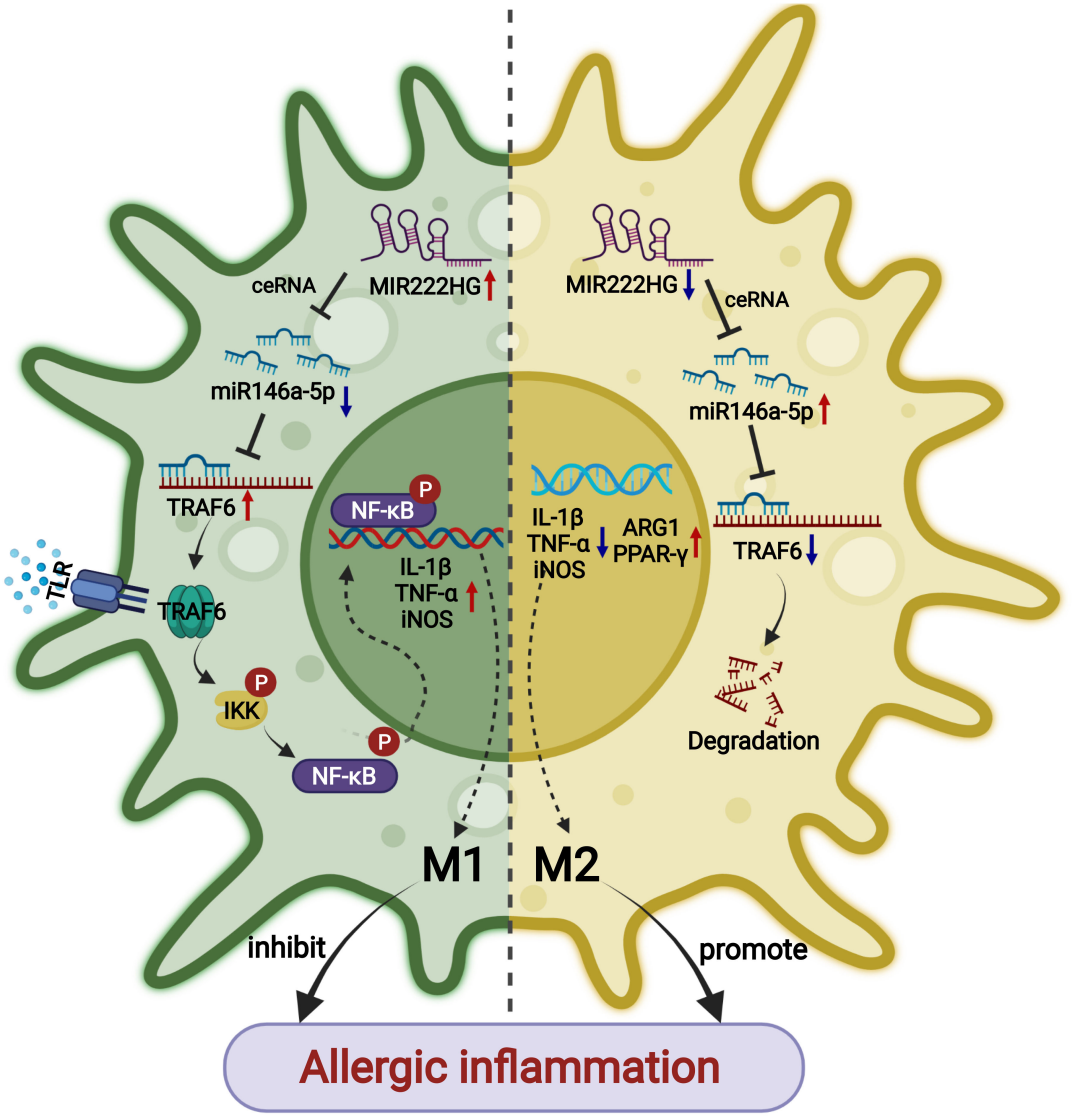
A schematic diagram showing that lncRNA-MIR222HG mediates macrophage polarization and suppresses AR by competing with miR146a-5p and activating TRAF6 and the IKK/IκB/P65 signaling pathway.

Collectively, the findings suggest that the intranasal administration of mir222hg–overexpressing lentivirus can alleviate allergic inflammation through the attenuation of M2 macrophage polarization in AR (**Figure 10**).

## 4 Discussion

Epidemiological observations have demonstrated that AR prevalence has increased dramatically over the last couple of decades, which cannot be explained by genetic variations in DNA (48, 49). Both reversible and irreversible epigenetic alterations, which alter the expression and function of genes without altering the genome nucleotide sequence, are deemed to be responsible for the rapid increase in AR prevalence (50–52). Regulatory ncRNAs, master post-transcriptional regulators in epigenetics (53), have attracted research attention because of their potential as biomarkers and their significance in the pathophysiology of allergic diseases (54–57). However, the molecular mechanisms responsible for the association of ncRNAs with AR are not comprehensively understood. Here, we conducted an *in silico* microarray analysis of allergic disease data from the GEO database (GSE165934) and validated that lncRNA-MIR222HG was significantly downregulated in both patients with AR and AR mice.

Our study results suggested that MIR222HG was highly conserved across species and matched with the ENSMUST00000143129.2 transcript of the Gm14636 gene (murine mir222hg), located on the murine chromosome X:19,023,220-19,033,471. miR-221/222 and the host gene MIR222HG, have been reported to regulate prostate cancer (13), atherosclerosis and hypertension (58), pulmonary arterial hypertension (59), and cell cycle re-entry (60). However, in addition to its function in regulating the expression of miR-221/222, MIR222HG was bioinformatically predicted to bind to miR146a-5p and share common MRE with TRAF6. Surprisingly, bioinformatic analyses revealed the same MRE overlap, AGUUCUCA, between murine mir222hg and Traf6. lncRNAs are competent in regulating mRNA by competitively binding miRNAs, whenever they contain common MRE (61, 62). qRT-PCR and FISH assay results suggested that MIR222HG/mir222hg is mainly localized in the cytoplasm of macrophages and likely functions as a ceRNA in macrophage inflammation.

Macrophages are highly dynamic and plastic immune cells in hematopoietic systems (63). In response to various microenvironmental signals, macrophages polarize to M1 phenotype and M2 phenotype and perform different functions. The two phenotypes mirror the Th1/Th2 states, respectively (6). The number of M2 phenotype macrophages is increased in the airway of allergic asthma patients and asthma mice, and is correlated with airway inflammation severity (64, 65). Furthermore, adoptive transfer of M2 macrophages promoted allergic inflammation (66). Consistent with the above studies, we found that the mRNA levels of the M2 macrophage markers (CD206 and ARG1) were elevated in AR patients. In our study, qRT-PCR and flow cytometry-based expression profiling of macrophage polarization markers in OVA-stimulated RAW264.7 cells suggested that allergen-OVA induces M2 polarization. Mir222hg expression decreased in OVA-stimulated macrophages in a dose-dependent manner. Concomitantly, we established that the post-transcriptional regulation along the mir222hg/miR146a-5p/Traf6 ceRNA axis could regulate macrophage polarization in the case of allergen stimulation.

MiR146a-5p, initially identified as an NF-κB-dependent miRNA, post-transcriptionally silences NF-κB pathway-related proteins, such as RelB, TLR-4, MyD88, IRAK1, and TRAF6, by binding to the 3′ UTR regions of the target mRNA (67–69). Emerging evidence suggests that miR146a-5p is enriched in IL-4-treated M2 macrophages (70) and promotes M2 polarization by inhibiting the NF-κB signaling pathway and inducing PPAR-γ (70–73). Consistent with the above studies, we found that miR146a-5p expression was significantly enriched in IL-4-treated RAW264.7 cells when compared with naïve and LPS-stimulated RAW264.7 cells. Mir222hg and miR146a-5p were co-localized in the cytoplasm. MiR146a-5p administration to the LPS/IL-4/OVA-treated RAW264.7 cells resulted in the downregulation of several M1 markers (CD86, IL-1β, TNF-α, and iNOS) and upregulation of M2 markers (CD206, ARG1, PPAR-γ), mediated *via* the inhibition of the Traf6/IKK/IκB/P65 signaling pathway. However, miR146a-5p reliant enrichment of CD206, ARG1, and PPAR-γ were abrogated accompanied by upregulation of CD86, IL-1β, TNF-α, and iNOS, upon mir222hg overexpression, indicating a credible interaction between mir222hg and miR146a-5p in macrophage polarization. Dual luciferase reporter gene assays verified this prediction, supporting our hypothesis that mir222hg functions as a ceRNA by competitively adsorbing the shared binding sequences of miR146a-5p, then sequestering miR146a-5p and regulating the downstream expression of Traf6.

Despite extensive research on allergen-specific immunotherapy and biologics behind AR in recent decades, such treatments have also failed to yield the desired efficacy owing to the great heterogeneity of AR patients (8). Knockdown and overexpression of mir222hg within the ceRNA network significantly reversed M1/M2 macrophage polarization caused by knockdown/overexpression of miR146a-5p. Furthermore, the intranasal administration of mir222hg-overexpressing lentivirus alleviated the allergic inflammation of AR through the attenuation of M2 macrophage polarization *in vivo*. These findings suggest that ncRNAs are potential therapeutic targets for AR. However, miR146a-5p targets multiple genes, thus causing a broader inflammatory response, leading to immune-related adverse effects in clinical trials, such as systemic inflammatory response syndrome and cytokine release syndrome (74, 75). Compared with miRNA mimics and anti-miRs, lncRNA-MIR222HG could specifically target macrophages owing to tissue specificity, thus striking a balance between efficacy and safety. Research on the novel MIR222HG-associated ceRNA network may open new avenues in AR therapy and facilitate the overcoming of traditional drug limitations.

It should, however, be noted that available experimental evidence could not directly demonstrate that MIR222HG affected AR pathogenesis by regulating macrophage polarization. In future studies we will further verify and evaluate the role of MIR222HG in macrophage-depletion AR model.

In conclusion, our study showed that lncRNA-MIR222HG attenuates M2 macrophage polarization in AR by decoying miR146a-5p to activate the TRAF6/IKK/IκB/P65 signaling pathway, and could act as a novel biomarker or potential therapeutic target for AR.

## Data availability statement

The original contributions presented in the study are included in the article/**Supplementary Material**. Further inquiries can be directed to the corresponding authors.

## Ethics statement

The studies involving human participants were reviewed and approved by the institutional ethical review board of Renmin Hospital of Wuhan University (approval WDRY2018-K052). Written informed consent to participate in this study was provided by the participants’ legal guardian/next of kin. The animal study was reviewed and approved by Institutional Animal Care and Animal Use Committee of the Renmin Hospital of Wuhan University (License No.: WDRM-20200602).

## Author contributions

SW, FL performed experiments and wrote the manuscript. YD and ZT designed the study and revised the manuscript. YT, LD and YH helped conduct the experiments, collected the clinical data and analyzed data. All authors contributed to the article and approved the submitted version.
