# Supplementary material for: MIR222HG attenuates macrophage M2 polarization and allergic inflammation in allergic rhinitis by targeting the miR146a-5p/TRAF6/NF-κB axis
Source: Front Immunol. 2023 May 2;14:1168920. doi: 10.3389/fimmu.2023.1168920 (PMC10185836; doi:10.3389/fimmu.2023.1168920)
Supplement: Supplementary file 1 [file DataSheet_1.docx]

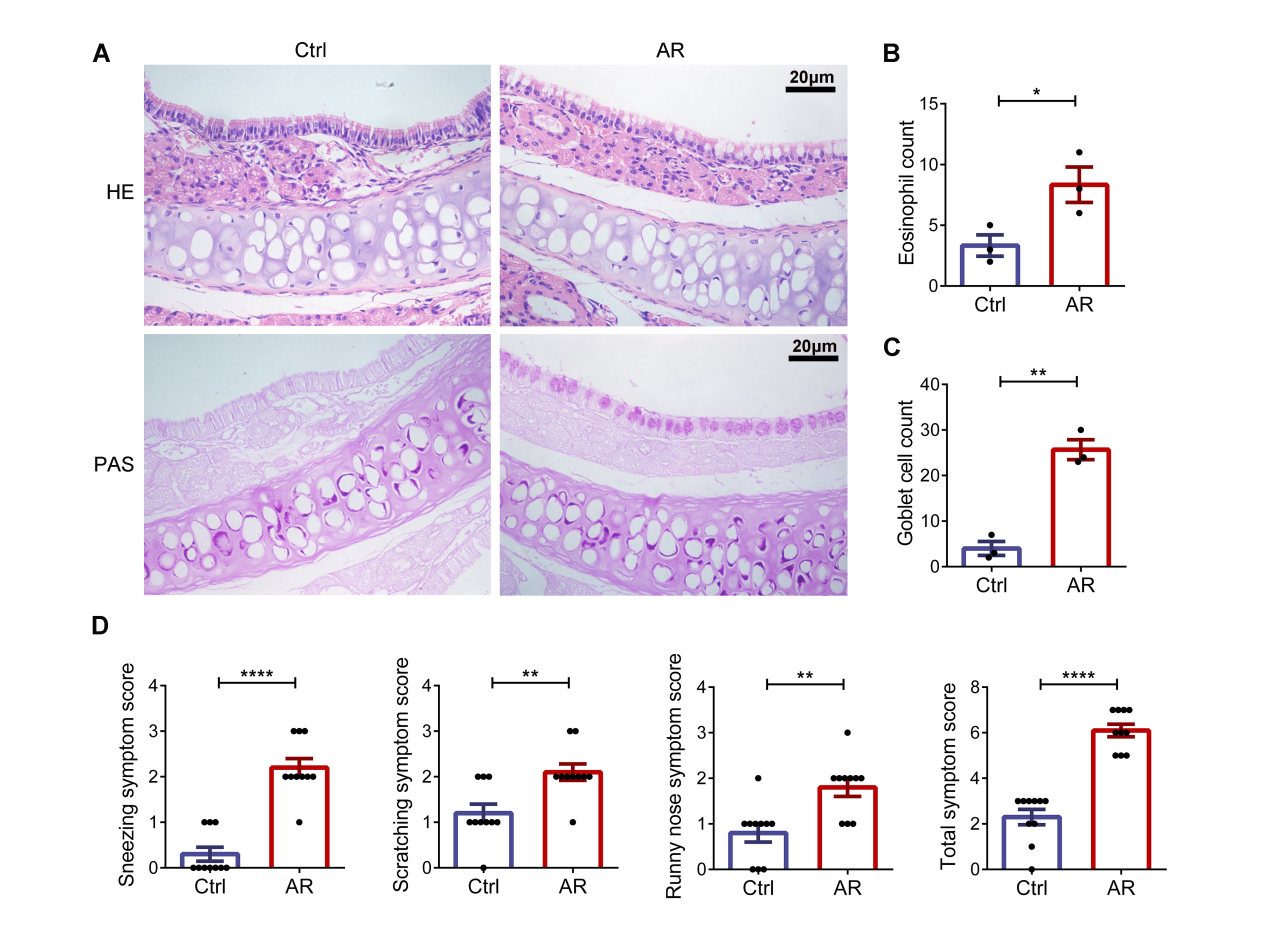


**Figure S1. The evaluation of AR animal model**

(A) Histopathologic changes of mice nasal mucosa as detected by HE and PAS staining. The original magnification was ×400. Scale bars, 20 μm. (B) The eosinophil count in nasal mucosa of mice in the AR and the control group. (C) The goblet cell count in nasal mucosa of mice in the AR and the control group. (D) The partial and total AR nasal symptom scores in AR mice and the control group. Symptom scores were defined as follows: 0 points: 0 sneezes, no runny nose, no nose scratching; 1 point: 1–3 sneezes, runny nose visible in the nasal opening, slight scratching of the nose several times; 2 points: 4 to 10 sneezes, nasal clearing over the nasal opening, repeated nose scratching with both paws; 3 points: >11 sneezes, runny face, scratching nose, rubbing around. A total score > 5 represents successful modeling. Each point represents data from one individual sample. Data are shown as the mean±SEMs (n= at least 3 samples per group). Data are merged from three independent experiments. Statistical significance was calculated by unpaired t-test. * p< 0.05, ** p< 0.01, **** p< 0.0001.
