# Supplementary material for: MIR222HG attenuates macrophage M2 polarization and allergic inflammation in allergic rhinitis by targeting the miR146a-5p/TRAF6/NF-κB axis
Source: Front Immunol. 2023 May 2;14:1168920. doi: 10.3389/fimmu.2023.1168920 (PMC10185836; doi:10.3389/fimmu.2023.1168920)
Supplement: Supplementary file 2 [file DataSheet_2.docx]

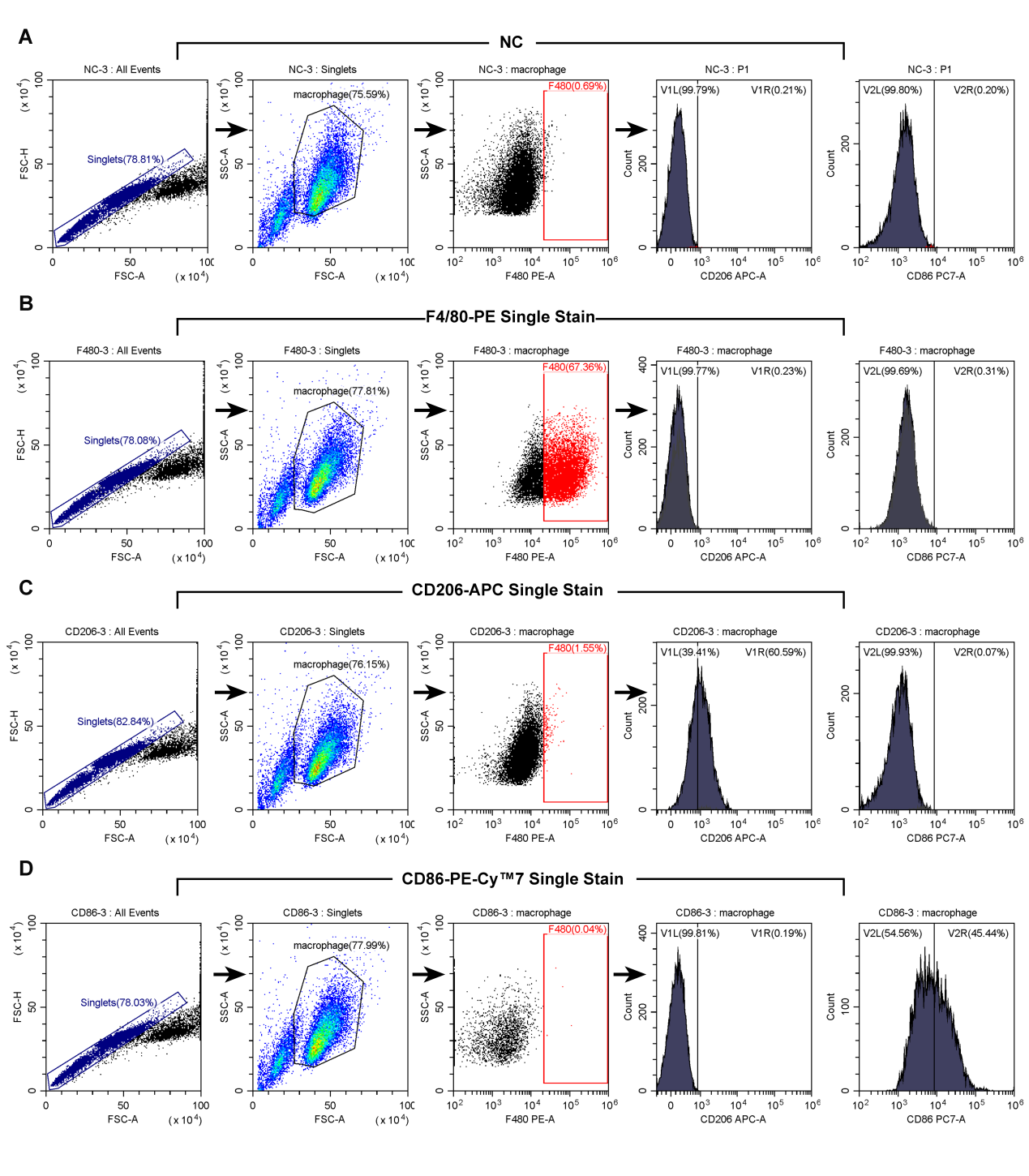


**Figure S2.** **Gating strategy for the identification of F4/80^+^CD86^+^ M1 macrophages and F4/80^+^CD206^+^ M2 macrophages**

A single cell suspension was prepared and stained with PE-conjugated anti-F4/80, PE-Cy™7-conjugated anti-CD86 and APC-conjugated anti-CD206. The area FSC-A against height FSC-H was used to discriminate doublets from singlets. Macrophages were identified by their scatter properties (FSC-A x SSC-A plot). (A) Nonspecific binding was checked within the sample by unstained cells (NC). (B-D) Single stained samples were used to compensate and correct spectral crosstalk of multicolor analyses. Representative flow cytometry plots showing gating strategy for unstained cells (A), F4/80-PE single stained samples (B), CD206-APC single stained samples (C) and CD86-PE-Cy™7 single stained samples (D).
