# Supplementary material for: MIR222HG attenuates macrophage M2 polarization and allergic inflammation in allergic rhinitis by targeting the miR146a-5p/TRAF6/NF-κB axis
Source: Front Immunol. 2023 May 2;14:1168920. doi: 10.3389/fimmu.2023.1168920 (PMC10185836; doi:10.3389/fimmu.2023.1168920)
Supplement: Supplementary file 3 [file DataSheet_3.docx]

**
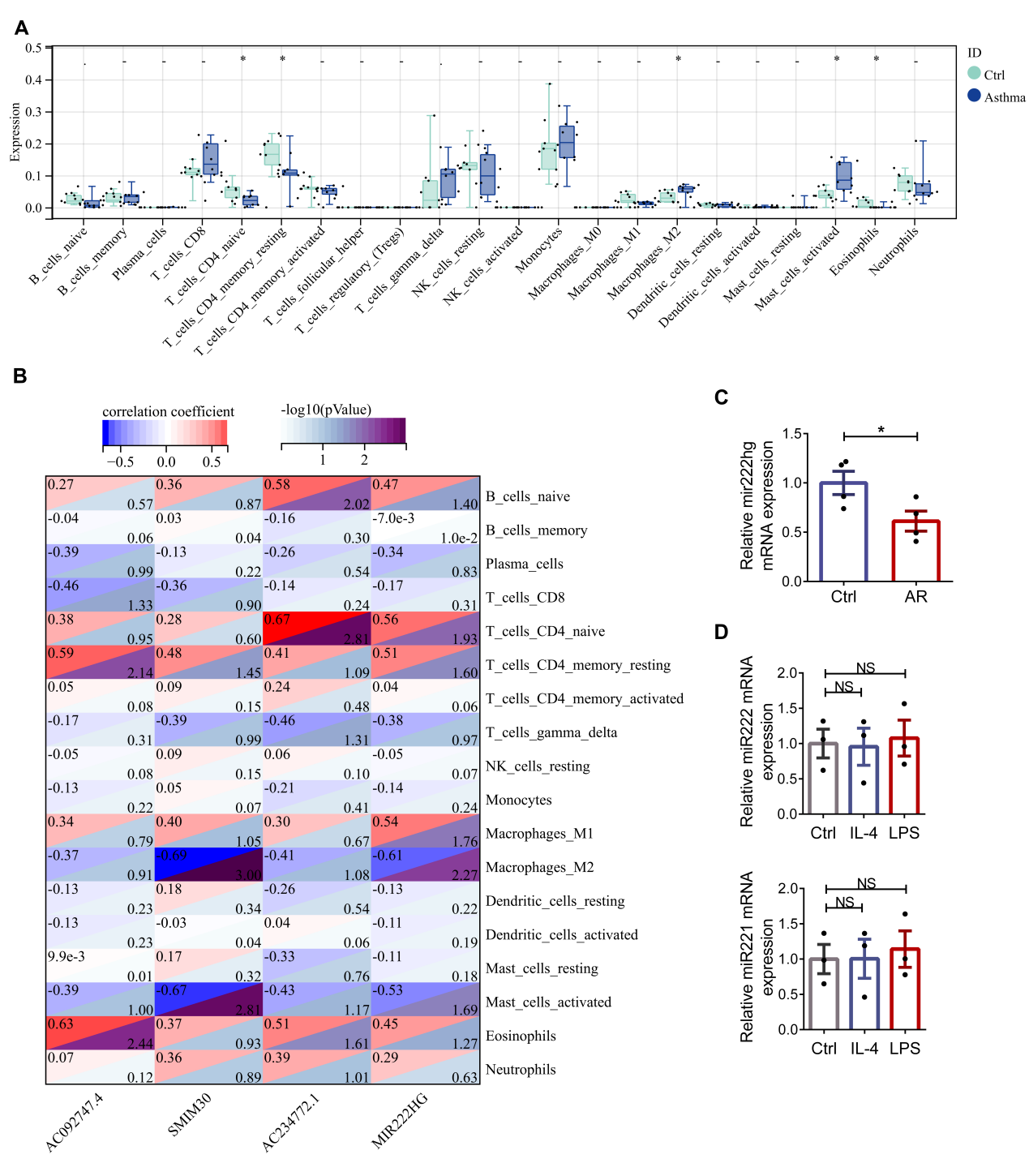
**

**Figure S3. Immune cell composition analyses and the mRNA expression of mir222hg and miR221/miR222**

(A) Immune cell composition analysis of microarray data (GSE165934). (B) Matrix correlation analysis between the expression of four highly conserved hub DE-lncRNAs and the ratios of immune cells. (C) The qRT-PCR was performed to detect mir222hg mRNA expression in macrophages isolated from the spleen of mice in the AR and the control group. (D) The qRT-qPCR showing miR221 and miR222 expression in LPS-induced M1 and IL-4-induced M2 macrophages. Each point represents data from one individual sample. Data are shown as the mean±SEMs (n= at least 3 samples per group). Data are merged from three independent experiments. Statistical significance was assessed by unpaired t-test for experiments comparing two groups appropriately, whereas two-way ANOVA followed by Sidak’s multiple comparisons test was used for comparisons between more than two groups. * p< 0.05, NS no significance.
