# Supplementary material for: MIR222HG attenuates macrophage M2 polarization and allergic inflammation in allergic rhinitis by targeting the miR146a-5p/TRAF6/NF-κB axis
Source: Front Immunol. 2023 May 2;14:1168920. doi: 10.3389/fimmu.2023.1168920 (PMC10185836; doi:10.3389/fimmu.2023.1168920)
Supplement: Supplementary file 4 [file DataSheet_4.docx]

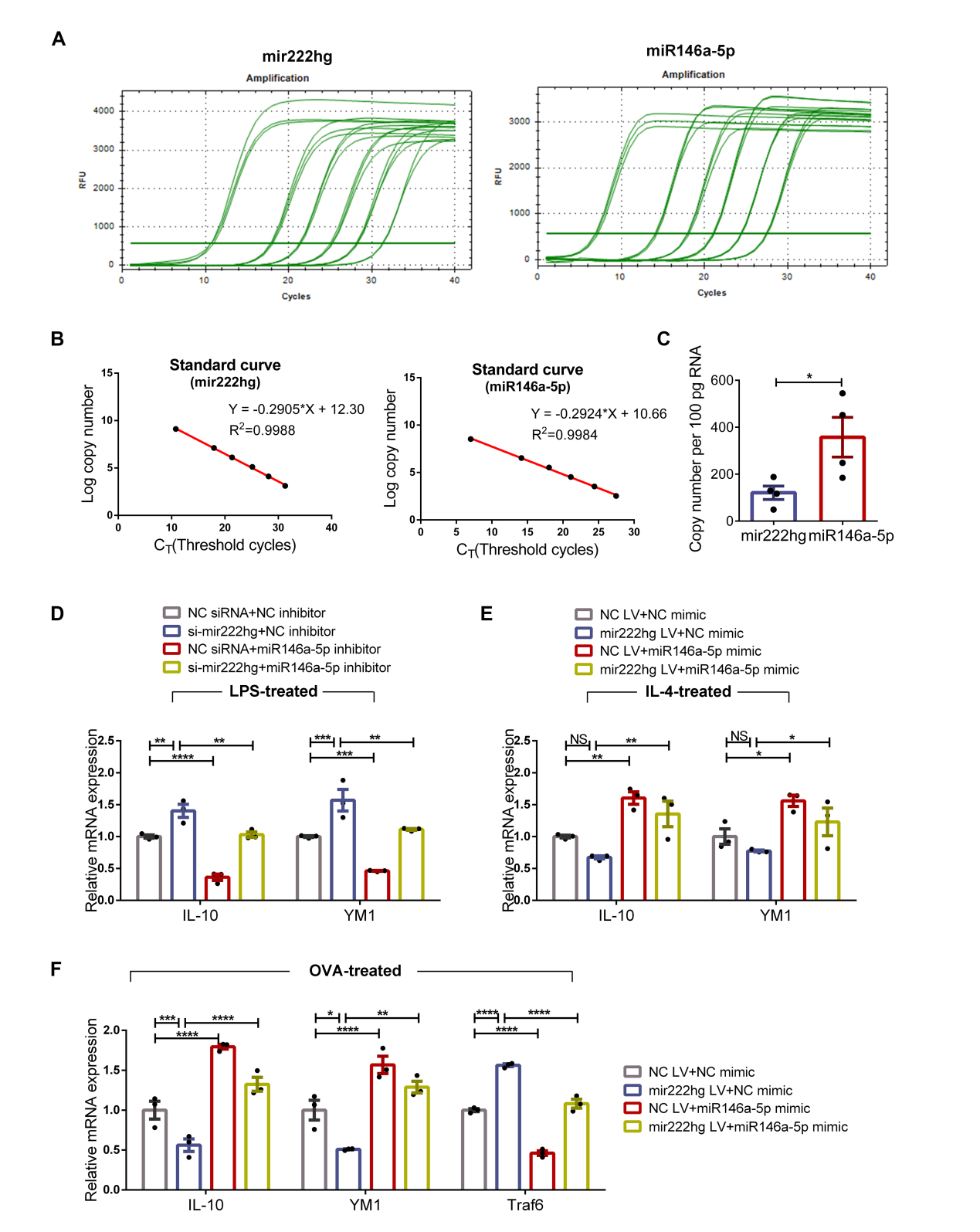


**Figure S4.** **The absolute expression of mir222hg/miR146a-5p in RAW264.7 cells and the relative mRNA expression of the M2 macrophage markers IL-10/YM1 in transfected RAW264.7 cells**

(A) Amplification curve of mir222hg and miR146a-5p in absolute quantification. (B) Standard curve of mir222hg and miR146a-5p in absolute quantification. (C) Absolute qRT-PCR assay was performed to detect copy number of mir222hg and miR146a-5p in 100 pg RNA of RAW264.7 cells. (D) RAW264.7 cells were transfected with NC siRNA + NC inhibitor, si-mir222hg + NC inhibitor, NC siRNA + miR146a-5p inhibitor, or si-mir222hg + miR146a-5p inhibitor, respectively. The above transfected RAW264.7 cells were then treated with 100 ng/ml LPS for 24 h. qRT-PCR was performed to detect IL-10 and YM1 relative mRNA expression. (E-F) RAW264.7 cells were transfected with NC LV + NC mimic, mir222hg LV + NC mimic, NC LV + miR146a-5p mimic, or mir222hg LV + miR146a-5p mimic. The transfected RAW264.7 cells were treated with 40 ng/ml IL-4 or 500 μg/ml OVA for 48 h. qRT-PCR was performed to detect IL-10, YM1 and Traf6 relative mRNA expression. Each point represents data from one individual sample. Data are shown as the mean±SEMs (n= at least 3 samples per group). Data are merged from three independent experiments. Statistical significance was assessed by unpaired t-test for experiments comparing two groups, whereas two-way ANOVA followed by Sidak’s multiple comparisons test was used for comparisons between more than two groups. * p< 0.05, ** p< 0.01, *** p< 0.001, **** p< 0.0001, NS no significance.
