# Supplementary material for: MIR222HG attenuates macrophage M2 polarization and allergic inflammation in allergic rhinitis by targeting the miR146a-5p/TRAF6/NF-κB axis
Source: Front Immunol. 2023 May 2;14:1168920. doi: 10.3389/fimmu.2023.1168920 (PMC10185836; doi:10.3389/fimmu.2023.1168920)
Supplement: Supplementary file 5 [file DataSheet_5.docx]

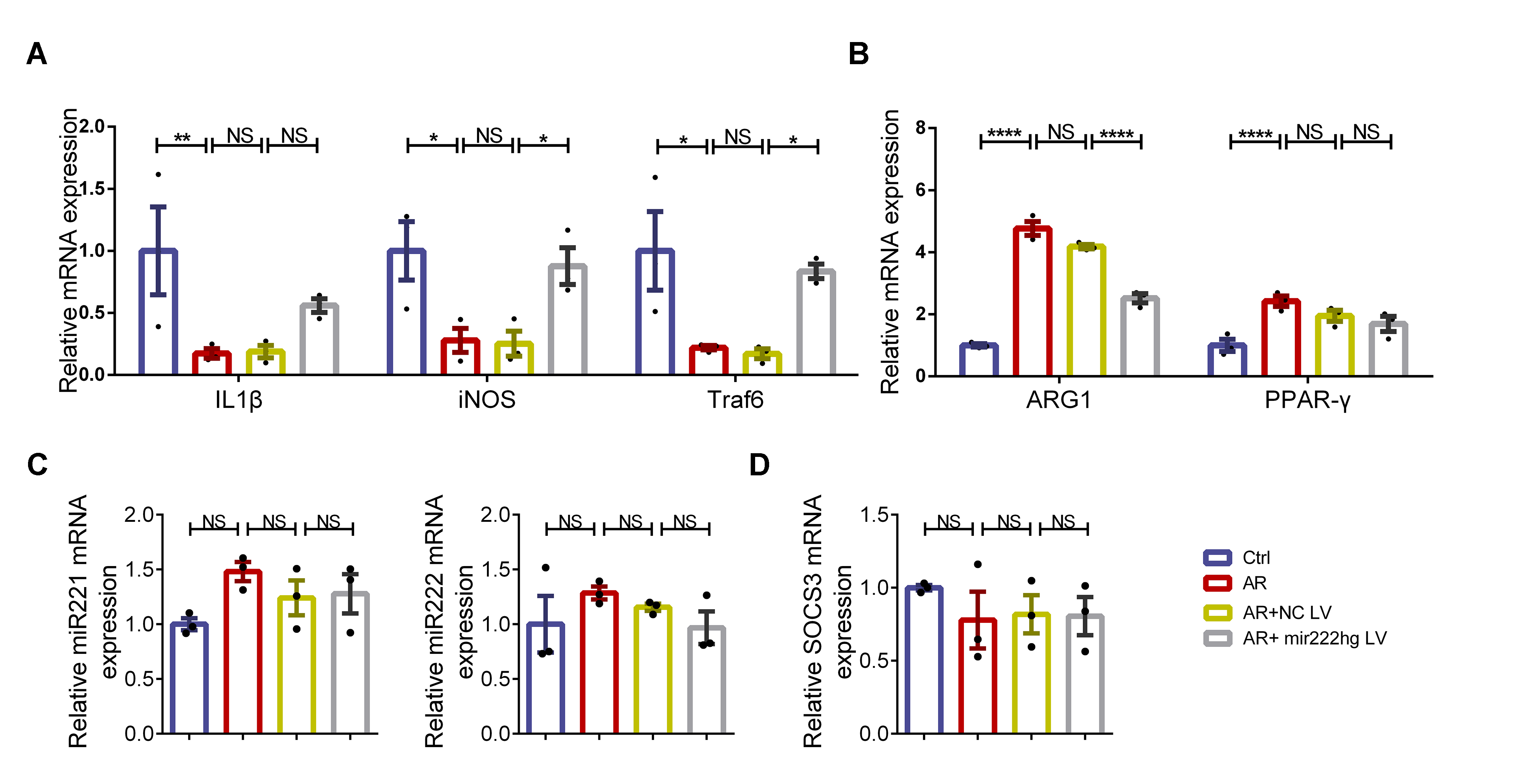


**Figure S5.** **Intranasal administration of mir222hg–overexpressing lentivirus attenuated M2 macrophage polarization in AR in an** **miR221/222 independent manner**

(A-B) qRT-PCR was performed to detect IL-1β, iNOS, Traf6, ARG1 and PPAR-γ relative mRNA expression. (C-D) qRT-PCR was performed to detect miR221, miR222 and Socs3 relative mRNA expression. Each point represents data from one individual sample. Data are shown as the mean±SEMs (n= 3 per group). Data are merged from three independent experiments. Statistical significance was assessed by two-way ANOVA followed by Sidak’s multiple comparisons test. * p< 0.05, ** p< 0.01, **** p< 0.0001, NS no significance.
